# Concordance between gambling disorder, alcohol use disorder, and other behavioural addictions provides evidence for a comprehensive approach to addictive behaviours: A multiple systematic review analysis

**DOI:** 10.1192/j.eurpsy.2026.12249

**Published:** 2026-08-12

**Authors:** Clément Vansteene, Daphnée Poupon, Andrea Czakó, Tamás Kurimay, Harshdeep S. Mangat, Ronald K. Ngetich, Zsolt Demetrovics, Philip Gorwood

**Affiliations:** 1 Universite Paris Cite, Institute of Psychiatry and Neuroscience of Paris (IPNP), INSERM U1266, Paris, France; 2 GHU Paris Psychiatrie et Neurosciences, Hopital Sainte-Anne (CMME), Paris, France; 3 Flinders University Institute for Mental Health and Wellbeing, College of Human Sciences and Culture, Flinders University, Bedford Park, SA, Australia; 4 Institute of Psychology, ELTE Eotvos Lorand University, Budapest, Hungary; 5 Centre of Excellence in Responsible Gaming, University of Gibraltar, Gibraltar, Gibraltar; 6 North-Buda Saint John Central Hospital, Buda Family Centered Mental Health Centre, Department of Psychiatry and Psychiatric Rehabilitation, Teaching Department of Semmelweis University, Budapest, Hungary; 7 Institute for Human Development, Aga Khan University, Nairobi, Kenya

**Keywords:** behavioural addictions, binge eating disorder, compulsive sexual behaviour disorder, gambling disorder, gaming disorder

## Abstract

**Background:**

Non-substance-related addictive behaviours have traditionally been treated separately from substance use disorders. However, the DSM-5 reclassification of gambling disorder within *Substance-Related and Addictive Disorders* has opened new avenues for research. This decision was justified by the similarities in the phenomenology, psychological, neurobiological, and genetic correlates across these disorders. Through a comprehensive systematic analysis, we aimed to identify factors associated with gambling disorder and examine their involvement in candidate behavioural addictions to assess the case for reclassification.

**Methods:**

Using gambling disorder as the gold standard, we identified 75 factors grouped into 9 categories to compare gambling disorder with alcohol use disorder (AUD) as a standard chemical addiction, gaming disorder, compulsive sexual behaviour disorder (CSBD), binge eating disorder (BED), compulsive buying–shopping disorder (CBSD), exercise addiction, and work addiction.

**Results:**

Data availability, compared to gambling disorder, was 100% for AUD; however, it was limited, ranging between 48% and 69% for gaming disorder, BED, and CSBD, and remained below 23% for other behaviours. The analyses confirmed a strong similarity between gambling disorder and alcohol use disorder (92.2%) and gaming disorder (98.9%), followed by CSBD (89.7%) and BED (84.9%). All other conditions showed weaker, although still high, congruence with gambling disorder (between 67% and 74% for the unweighted concordance rates).

**Conclusions:**

Our findings reaffirm AUD, gambling, and gaming disorder as addictive disorders and provide evidence that the studied candidates could be considered as behavioural addictions, especially binge eating disorder and CSBD.

## Introduction

Classification decisions in international diagnostic manuals are consequential: placement of a disorder within a particular chapter influences clinical recognition and reimbursement [1], shapes the pharmaceutical and commercial landscape [2], drives research priorities [3], and frames how the spectrum of mental disorders is presented worldwide [4]. Each new edition invites debate about whether particular syndromes should be included or excluded from given categories [5].

Until recently, major diagnostic systems restricted the term *addiction* to problems involving psychoactive substances, with diagnostic terminology focusing on dependence and abuse [6]. Over the past decade, however, the category has been expanded to encompass non-substance-use-related (behavioural) addictions. A key milestone was the DSM-5 decision (2013) to move gambling disorder from *Impulse-Control Disorders* to *Substance-Related and Addictive Disorders* [7]. The World Health Organization ICD-11 subsequently grouped gambling disorder within *Disorders due to Addictive Behaviours* and added gaming disorder as a second formally recognised behavioural addiction [8].

Gambling disorder was indeed the first behavioural condition reclassified as an addictive disorder, supported by robust evidence of parallels with substance use disorders across clinical phenomenology [9], epidemiology [10], cognitive and decision-making deficits [11, 12], biological and neurophysiological findings [13], neuroimaging [14], genetic/twin studies [15], and treatment processes [16]. The reclassification of gambling disorder in the DSM-5 was based on the convergence of several constitutive features of addiction: loss of control, preoccupation and craving, escalating engagement, irritability upon cessation, and persistence despite adverse consequences [17]. These clinical similarities extend, at the biological and epidemiological levels, to shared psychiatric comorbidities, common genetic contributions, and converging neurobiological mechanisms within the reward circuitry [18]. The recognition of analogous patterns in other repetitive behaviours has motivated their progressive integration within a broadened but still contested addiction framework.

Research on behavioural addictions has since followed a heterogeneous trajectory rather than an undifferentiated expansion: between 1995 and 2019, the field grew by an overall 130%, driven primarily by information- and communication-technology-related disorders, gaming and food addiction, while gambling research increased by only 99.4% [19]. On PubMed, annual output stabilised at around 450–500 publications between 2022 and 2024, before rising above 600 in 2025 [20]. Prevalence studies further show meaningful pooled rates: for example, a pandemic-era meta-analysis of 94 studies across 40 countries (*n* = 237,657) reported pooled prevalence rates for gaming (5.3%), exercise (7%), gambling (7.2%), shopping (7.2%), sex (9.4%), and food (21%) addiction [21]. Together, these trends justify close examination of which additional behaviours, if any, warrant formal inclusion alongside substance use disorders and gambling disorder [22].

Not every proposed behavioural addiction is equally likely to meet the evidentiary threshold for reclassification. A defensible proposal should rest on converging criteria with an adequate, replicated empirical base across clinical, epidemiological, cognitive, neuroscientific, and genetic domains, with consistent findings across those domains. Using gambling disorder reclassification as precedent and alcohol use disorder (AUD) as a benchmark, we systematically compared the evidence for gambling disorder against that available for six frequently discussed candidate behavioural addictions: gaming disorder, compulsive sexual behaviour disorder (CSBD), binge-eating disorder (BED), compulsive buying–shopping disorder, exercise addiction, and work addiction.

Empirical clustering studies and co-occurrence analyses suggest both coherence and heterogeneity across behavioural addictions. Some data indicate disorder-specific profiles, while other findings reveal shared vulnerability clusters spanning multiple behaviours. For example, a large cluster analysis of ~6,000 respondents identified seven clusters of addictive behaviours: five were mainly driven by a unique addictive disorder, yet all seven overlapped, at least partially, with the five disorders – a pattern highlighting both communality and specificity [23]. The two clusters with broad cross-disorder co-occurrence were a “polyaddicted” profile combining alcohol, tobacco, cannabis, gambling, excessive eating, work addiction, and a cluster centred on problematic gaming, with marked overlap with tobacco, excessive eating, and work addiction.

Over recent decades, research has repeatedly drawn attention to links among these disorders, including similarities in symptomatology, high co-occurrence rates, and shared aetiological factors [6, 24, 25]. However, no comprehensive multi-domain synthesis has systematically compared behavioural candidates against a substance-use benchmark, limiting judgements about whether known addiction markers generalise to these behaviours.

The present study addresses this gap. We aimed to evaluate the availability, nature, and strength of evidence for a shared addictive basis across gambling disorder, AUD, and six candidate behavioural disorders. To do so, we developed and applied a multi-domain framework to assess reproducibility, using a PRISMA approach and searching for convergence between the two teams involved whenever a decision was based on factors other than data alone. Specifically, we (1) identified the most relevant empirical domains; (2) mapped and scored the available literature for each condition across those domains; and (3) synthesised whether the evidence supports conceptualising each candidate as an addictive disorder, as was done for gambling disorder. Our aim was to provide a transparent evidence map to inform diagnostic debate and guide research priorities.

## Methods

Gambling disorder, as the longest-standing and most extensively studied behavioural addiction, was defined as the reference disorder. Based on the systematic review of the literature on gambling disorder, we identified the factors associated with gambling disorder and used these as a reference for the next stage. Further analyses aimed to assess (1) to what extent evidence for the identified factors was available on the selected other behavioural addictions (i.e., data availability); (2) to what extent direction of available evidence on the selected behavioural addiction and on gambling disorder corresponded (i.e., level of concordance); and (3) how strong this evidence was (i.e., weighted concordance). Additionally, besides the selected behavioural addictions, AUD was analysed as the gold standard, representing substance use disorders in this comparative analysis.

Gaming disorder was the first included not only because of being a widely examined phenomenon but also because of being the second disorder recognised in the ICD-11 as an addictive disorder. We also included two disorders, CSBD and BED, which are recognised disorders in the ICD-11 but not as addictive disorders, even though they are often referred to as having various addictive features. Finally, we included three further phenomena, compulsive buying–shopping disorder, exercise addiction, and work addiction, which are commonly discussed as addictive disorders, but are not included in the diagnostic systems. Internet addiction (or problematic internet use) [26] and social media addiction [27] were considered, but were finally not included in the current analyses because of their less specific umbrella nature. The six candidate entities were selected through an independent, blinded procedure conducted in parallel by the two research teams, on the basis of the frequency of the disorders and the density of the related literature; the two resulting lists were then compared, and consensus was reached on the final selection. A degree of subjectivity nonetheless remains inherent to this process.

### Search strategy

A systematic search was conducted for each behavioural addiction using four databases: PubMed, PsycINFO, Scopus, and Web of Science. Searches were limited to peer-reviewed meta-analyses and systematic reviews published in English from 2000 onwards. The review was carried out and reported according to the Preferred Reporting Items for Systematic Reviews and Meta-Analyses (PRISMA) guidelines [28]. The checklist is available in the Supplementary Table S1.

For gambling disorder, the search was conducted on 6 January 2022. The following search string was used in PubMed: gambl*[title/abstract]. Filters were applied to select meta-analyses and systematic reviews published from 2000 onwards. Search strings used in other databases are detailed in Supplementary Table S2. For the other behavioural addictions, searches were conducted on 29 June 2022 and updated on 20 January 2026. Details are provided in Supplementary Table S2.

### Study selection

After removing duplicates, studies were screened based on title and abstract. Then, the full texts of the preselected references were assessed. Two authors independently screened the references for gambling disorder and assessed the full-text articles. Inclusion criteria were (a) peer-reviewed meta-analyses or systematic reviews (b) with results relevant to our research question (i.e., related to factors associated with the addiction), (c) written in English, and (d) published from 2000 onwards.

### Level of evidence assessment

The level of evidence of each paper was assessed using the Scottish Intercollegiate Guidelines Network (SIGN) guidelines [29], as suggested by the European Psychiatric Association (EPA) guidance committee [30]. The level of evidence was composed of a number indicating the type of review (1 for meta-analyses, 2 for systematic reviews) and a code indicating the risk of bias (“++,” “+,” or “–,” indicating low, medium, or high risk of bias, respectively). The risk of bias was evaluated through five criteria, investigating whether: (1) the study addresses an appropriate and clearly focused question; (2) a description of the methodology used is included; (3) the literature search is sufficiently rigorous to identify all the relevant studies; (4) study quality is assessed and taken into account; and (5) there are enough similarities between the studies selected to make combining them reasonable. Each item was rated on a scale from 0 to 1. The total risk of bias score between 0 and 5 was computed by adding the ratings of each of the five levels of evidence assessment criteria. The following risk of bias codes were assigned to each study: “++” for those with a risk of bias score of 5, “+” for those with a risk of bias score of 3 or 4, and “–” for those with a risk of bias score between 0 and 2. Combining the number indicating the type of the review and the risk of bias codes, one of the following levels of evidence was assigned to each study: 1++, 1+, 1–, 2++, 2+, or 2–. For example, 1++ indicates a high-quality meta-analysis with a low risk of bias.

### Data extraction

For each selected article, the following data were extracted: the type of review (meta-analysis or systematic review), the factors evaluated, and the results obtained. As a first step, two authors independently extracted the data from the studies on gambling disorder, listing the evaluated factors for each included review. In case of discrepancy, gaps were resolved in a face-to-face meeting between the two raters.

### The case of alcohol use disorder

AUD was included as an example of substance use disorder, serving as a reference. AUD was selected among substance use disorders based on its high population-level prevalence – particularly in the WHO European Region where both research teams are based [31] – and its extensive epidemiological documentation, which together provide the most robust dataset for a calibrating comparator [32]. Considering that the literature on alcohol use disorder is much broader than on behavioural addictions, and research methods are generally more accurate (e.g., direct analyses in the global population), a slightly different methodology was applied. Instead of systematic reviews, we prioritised recent epidemiological studies and extracted data related to factors found for gambling disorder. When no information was available in epidemiological studies, meta-analyses or systematic reviews were sought. The level of evidence category attributed to epidemiological studies was 1+++, that is, ranked higher than meta-analyses with low risk of bias, while levels of evidence for meta-analyses and systematic reviews were computed as detailed above.

### Data synthesis

The first part of data synthesis included establishing the list of factors found for gambling disorder (*N* = 75). This was done before extracting data for other behavioural addictions. To do so, we attributed to each factor a category in order to gather similar factors extracted from different articles and different addictions. Nine main categories were identified, namely “global environment” (comprising 16 individual factors), “psychiatric comorbidity” (12 factors), “addictive comorbidity” (7 factors), “somatic comorbidity” (3 factors), “personality and temperament” (11 factors), “cognitive characteristics” (14 factors), “emotional dysregulation” (6 factors), “genetics” (2 factors), and “brain assessments” (4 factors).

As a follow-up step, a comparison dataset was built (see Supplementary Table S3), including the following information: a first column listing all factors, followed by two columns for each addiction. In the first of the two columns, we reported the result (i.e., whether the factor is positively or negatively associated with the addiction, or results were inconclusive). The second one indicates the level of evidence supporting the study’s result. When several studies presented results on the same factor, we applied the highest level of evidence. If results from different studies did not concur, we prioritised (1) the study with the highest level of evidence, and (2) the most recent study, on the rationale that more recent systematic reviews and meta-analyses typically incorporate a larger and more up-to-date set of primary studies and therefore provide a more comprehensive synthesis. A third step (favouring significant over non-significant findings) was planned as a final tie-breaker only if discrepancies persisted after considering evidence level and recency, based on the assumption that non-significant findings may sometimes reflect insufficient statistical power. In practice, this final step was never required.

We quantified the similarity between each behavioural addiction and gambling disorder using three complementary metrics: (1) *data availability*, (2) *unweighted concordance*, and (3) *weighted concordance.* All metrics were first calculated within each of the nine categories of factors, after which an overall score was derived using metric-specific procedures (see below).

#### Data availability

For each addiction and each category, a data availability ratio was computed as the proportion of factors for which at least one study (of any level of evidence) was identified, relative to the total number of factors identified for gambling disorder. A global data availability ratio was calculated similarly across all factors.

#### Unweighted concordance

Unweighted concordance captured the directional agreement between each addiction and gambling disorder. For each addiction and category, we calculated the proportion of comparable factors showing concordant results (i.e., same direction of association as in gambling disorder), excluding factors with no available data. A global unweighted concordance ratio was obtained by averaging the category-specific concordance ratios across categories with available data.

#### Weighted concordance

To account for differences in the strength of evidence, we computed weighted concordance ratios. Each factor was assigned a numerical weight based on its level of evidence (1+++ = 6, 1++ = 5, 1+ = 4, 2++ = 3, 2+ = 2, 1−/2− = 1).

For each addiction and category, weighted sums were calculated separately for factors with positive, negative, and inconclusive associations, and aggregated at the category level. For gambling disorder (reference condition), corresponding weighted sums were computed for each comparison addiction, excluding factors lacking data in that addiction to ensure comparability.

Weighted concordance ratios were then obtained by dividing the weighted sum for each outcome type (positive, negative, and inconclusive) in the addiction by the corresponding sum in gambling disorder. Global weighted concordance ratios were obtained by averaging the category-specific weighted concordance ratios across categories with available data.

### A priori and post-hoc steps

The selection of disorders, search strategy, eligibility criteria, evidence-rating framework (SIGN), concordance metrics, and rules for resolving discrepant findings were specified before data extraction. In contrast, the final list of 75 factors and their grouping into 9 domains emerged inductively from the gambling disorder literature during data extraction and synthesis, based on both empirical patterns and expert consensus between the two research teams. Similarly, the exclusion of internet and social media addiction, as well as the operational weighting of evidence levels in the weighted concordance analyses, were finalised during the analytical phase.

## Results

### Study characteristics

The literature search identified 7,606 studies, 246 of which met the inclusion criteria (Figure 1), in addition to the 49 studies included for AUD. Among the 246 included studies, 58 discussed gambling disorder, 65 gaming disorder, 64 BED, 30 CSBD, 11 compulsive buying–shopping disorder, 13 exercise addiction, and 4 work addiction. Flow charts for each behavioural addiction are available in Supplementary Figure S1. The identified manuscripts included in the analysis for each factor and disorder are listed in Supplementary Table S4.

**Figure 1.**
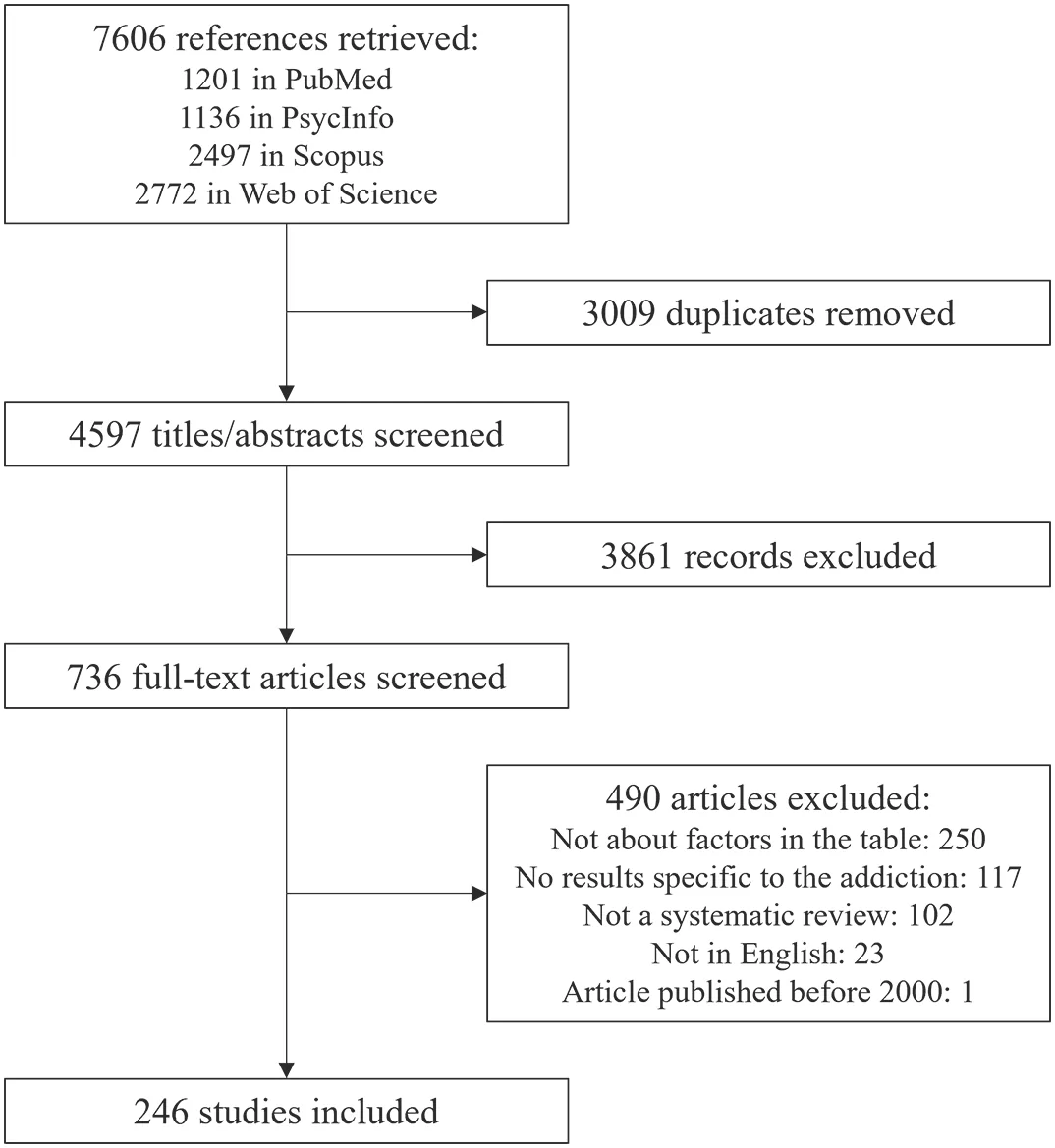
PRISMA flow chart of included studies. Figure 1. Long description.

### Data availability

The search on gambling disorder yielded 75 factors that we grouped into 9 main categories, as presented above. The overall data availability for each disorder is presented in Table 1. All but three of these factors, all of which belong to the brain-assessments category, could be identified in the AUD-related studies as well, resulting in 100% of data availability. The second highest level of data availability was found in the case of gaming disorder (69.3%), followed by BED (54.7%) and CSBD (48.0%). Data availability remained below 25% in the case of work addiction (20.0%), exercise addiction (20.0%), and compulsive buying–shopping disorder (22.7%). In the case of gaming disorder, relatively higher data availability was observed regarding emotional dysregulation studies (100.0%), personality and temperament (81.8%), and cognitive characteristics (71.4%), and lower in the case of addictive (28.6%) and psychiatric (33.3%) comorbidities. Regarding BED, despite the much lower data availability rates, at least some studies were available in each main category, which was also true for eight out of the nine categories in relation to CSBD. Data availability was lower for the other three disorders (see Figure 2A for data availability for each category and disorder).

**Table 1.** Overall data availability, level of unweighted concordance, and level of weighted concordance in AUD and candidate behavioural addictions compared to gambling disorder Table 1. Long description.

	Alcohol use disorder (%)	Gaming disorder (%)	Binge eating disorder (%)	Compulsive sexual behaviour disorder (%)	Work addiction (%)	Exercise addiction (%)	Compulsive buying–shopping disorder (%)
Data availability	100.0	69.3	54.7	48.0	20.0	20.0	22.7
Unweighted concordance							
Congruent	92.2	98.9	84.9	89.7	70.7	66.7	73.8
Incongruent	0.0	0.0	4.0	0.0	3.3	4.8	6.3
Inconclusive	7.8	1.1	11.1	10.3	26.0	28.6	20.0
Weighted concordance							
Congruent	119.3	85.2	75.2	50.1	84.5	60.2.	45.2
Incongruent	0.0	0.0	1.9	0.0	3.3	3.9	6.3
Inconclusive	9.9	0.7	7.7	4.9	24.5	22.9	14.1

**Figure 2.**
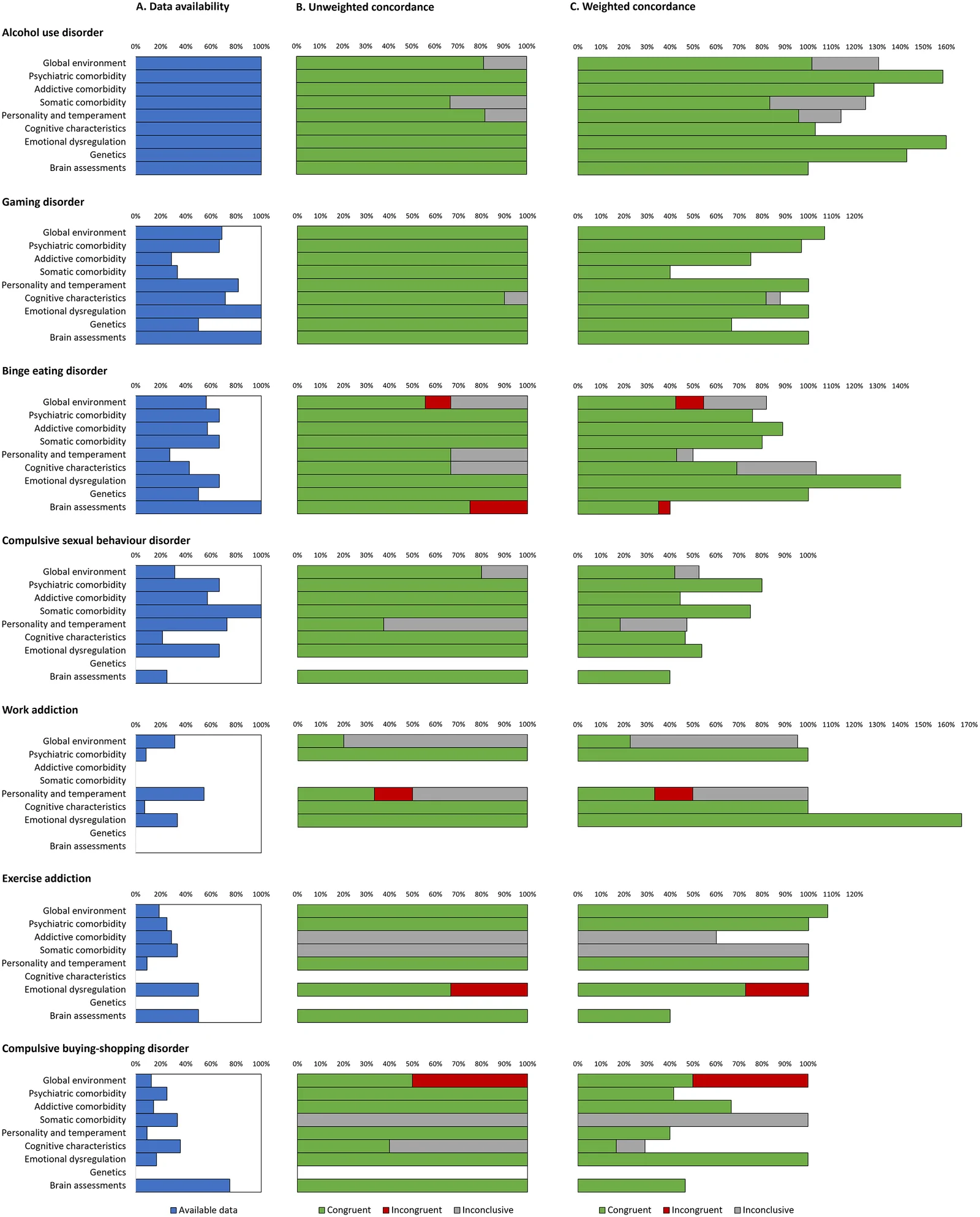
Data availability (A) and their concordance (unweighted [B] and weighted for the level of evidence [C]) in alcohol use disorder (reference of substance use disorder) and six possible addictive disorders, of 75 factors, merged in nine main categories, characterizing gambling disorder (standard of behavioural addiction). *Note:* The availability of data (2A) ranges from 0% to 100%. This is presented for each dimension and each possible addictive disorder in blue rectangles. Concordance rates are located on the right part of the figure, either as unweighted percentages (2B) or weighted percentages according to the level of evidence (2C). Green rectangles correspond to data that aligns with observations relating to gambling disorder (e.g., an excess of males), and grey rectangles correspond to inconclusive results (e.g., data not available). Red rectangles indicate results that are the opposite of those observed in gambling disorder (e.g., an excess of females). Concordance rates of alcohol use disorder could exceed 100% as the only disorder for which epidemiological data were available. This is because the confidence level of epidemiological data is higher than that of systematic reviews and meta-analyses. Figure 2. Long description.

### Unweighted concordance

Narrowing the subsequent analyses to the comparison of available data, we examined whether the studies showed the same or opposite effect as gambling disorder along each factor, or did not reveal conclusive results (see Figure 2B for category-specific unweighted concordance and Table 1 for the overall level of unweighted concordance). These results showed that the vast majority of the studies provided evidence that the examined factors had a similar association with the different behavioural addictions and AUD. The overall unweighted concordance was 98.9% for gaming disorder, 92.2% for AUD, 89.7% for CSBD, 84.9% for BED, 73.8% for compulsive buying–shopping disorder, 70.7% for work addiction, and 66.7% for exercise addiction. On the other hand, incongruent results remained below 7%. In the case of AUD, 100% concordance was observed in five out of the nine categories, while in the other four categories, this value ranged between 66.7% and 81.8%. It is important to mention that all the remaining results were inconclusive; thus, none of the evidence showed the opposite direction compared to gambling disorder. Very similar tendencies were found in the case of the six behavioural addictions as well. Incongruent results were only found in the case of compulsive buying–shopping disorder (opposite gender effect), exercise addiction (opposite self-esteem effect), work addiction (opposite extraversion effect), and BED (opposite gender effect and one of the four brain-related factors). The prevalence of inconclusive findings was the lowest for gaming disorder (1.1%). It ranged between 7.8% and 10.3% for AUD, BED, and CSBD, and between 20.0% and 28.6% for exercise addiction, compulsive buying–shopping disorder, and work addiction.

### Weighted concordance

Weighted concordance results showed that, with the exception of AUD, the level of evidence available to support the examined factors’ effect on the different behavioural addictions is lower than in the case of gambling disorder. As an additional higher level of evidence was introduced in the case of AUD, the score was 100.0% for brain assessments, and above 100% for the other eight categories. The mean weighted concordance between AUD and gambling disorder was 119.3%. AUD was followed by gaming disorder (85.2%), work addiction (84.5%), BED (75.2%), and exercise addiction (60.2%). It was around 50% for CSBD (50.1%) and compulsive buying–shopping disorder (45.2%; see Figure 2C for the category-specific weighted concordance and Table 1 for the overall level of weighted concordance).

## Discussion

Relying on close to 300 relevant systematic reviews and meta-analyses on AUD (used here as the gold standard for substance use disorder), gambling disorder (the gold standard for behavioural addictions), and six candidate behavioural addictions (concerning gaming, binge eating, sex, work, exercise, and shopping), we confirmed a strong proximity between AUD and gambling disorder, followed by gaming disorder. From the other investigated conditions, CSBD and BED also had a large evidence base that mostly supports shared associated factors. Compulsive buying–shopping disorder, exercise addiction, and work addiction have been less extensively studied, but the existing evidence shows moderate to high levels of congruence with gambling disorder. Beyond these findings, the specific contribution of our approach lies in bringing together a wide range of behavioural addictions within a single comparative framework – not to quantify the volume of literature devoted to each, but to confront their empirical profiles with the gold standard of gambling disorder, and ultimately with that of a well-characterised substance-related addiction, AUD.

Neurobiologically and psychologically, gaming disorder and BED engage mechanisms implicated in substance use, including dopaminergic reward pathways, impaired impulse control, and cue-reactivity [33, 34]. This explains why incentive-salience and reward-deficiency models developed for drug addiction have been applied to gaming, gambling, and food compulsion alike [34]. The WHO’s recognition of gaming disorder as a *Disorder due to Addictive Behaviours* further reflects this overlap [8]. BED is also often conceptualised as an addictive phenotype, since high-calorie foods can provoke craving and tolerance-like effects akin to drugs. CSBD also shares features with substance use and gambling disorder, including craving and loss of control [35]. Cognitive commonalities are also documented: attentional bias and both motor and reflective impulsivity have been observed in at least two of four disorders (gambling, gaming, binge eating, and sex addictions), providing cross-disorder evidence of shared mechanisms [36].

These results suggest that the evidence generated by the present analysis – based on a set of 75 factors identified in the literature – is consistent with the view that these behaviours are behavioural addictions. However, this conclusion should be interpreted with caution, as several clinically relevant dimensions were not captured in the present framework due to the lack of available studies. In particular, factors such as functional impairment or disability, the need for care, and responsiveness to treatment may carry substantial weight in classification decisions. Future analyses integrating these dimensions would be needed to draw more definitive conclusions. There is a relatively higher proportion of inconclusive results, which may reflect both addiction-like processes and mechanisms outside our extraction framework, and also highlight the need for further research. Indeed, the 75 distinct factors grouped into nine domains were derived from gambling disorder studies and may omit features more relevant to shopping, exercise, or work addiction (e.g., body-image goals and perfectionism). These results align with the view that current evidence may be insufficient to justify classifying these behaviours alongside substances.

A key caveat is that data availability limits the conclusions that can be drawn. Syndromes with larger or older literatures (e.g., AUD, gambling disorder, BED, and gaming disorder) are more likely to show convergent, multi-level evidence, partly because they have been studied more intensively with sophisticated methods. By contrast, behavioural addictions with fewer studies may appear divergent or have inconclusive results, not because they are fundamentally different, but because rigorous, multi-domain studies are lacking. This asymmetry particularly concerns work addiction, exercise addiction, and compulsive buying–shopping disorder, whose limited empirical bases may weigh on the comparisons we draw. Thus, the absence of evidence for similarity to substance addictions should not be mistaken for evidence of absence.

Some limitations of this review need to be acknowledged. First, we restricted the analysis to six phenomena (gaming, eating, sex, buying, working, and exercise), although new candidates continue to be proposed – a proliferation some have described as a “plague of our era” [37]. Notably, the six examined here are the very ones highlighted in that reference. Second, the number and granularity of the factors and corresponding items were not determined before the study, but emerged from the review of the existing empirical literature. The final grouping and number of items were based on both the literature and the authors’ expertise, and therefore inevitably involved a degree of subjectivity, particularly regarding categorisation and methodological choices. It is possible that incorporating other perspectives, such as broader expert consensus or more objective, predefined criteria, could have led to a different evaluation framework. Accordingly, the methodology applied here could be further refined and standardised in future studies. Third, the field lacks rigorous prospective data on *stability* and *functional impairment*, which are generally regarded as essential criteria for defining a pathological condition [37]. A 5-year longitudinal study [38] that examined the natural course and impact of several behavioural addictions found that excessive involvement in the targeted behaviours was often transient. Yet, not all addictive disorders – including substance-related ones – are stable, and impairment requirements can be added to diagnostic definitions. Importantly, the purpose of this review was to assess similarities across behavioural addictions, not to propose diagnostic criteria. Fourth, heterogeneity complicates comparisons. Standardised diagnostic criteria are absent, making cohorts difficult to compare across behaviours. Heterogeneity is also evident within disorders. For example, CSBD has been variably described as sex addiction, sexual compulsivity, or sexual impulsivity [39, 40], rendering a single definition premature [41]. A recent review concluded that evidence from epidemiology, phenomenology, mental-health assessment, and neurobiology is required to resolve such definitional issues [42] – the rationale underpinning the present study. Fifth, our choice to study AUD, rather than another substance use disorder, via primary epidemiological studies rather than systematic reviews or meta-analyses may be criticised, since a different approach for AUD could bias detectable risk factors. We judged, however, that systematic reviews and meta-analyses of associated risk factors are a poorer proxy for population-level epidemiology. Given the abundance of large, high-quality datasets on alcohol (e.g., NESARC), including minority populations, direct epidemiological data were considered preferable.

Designating a behaviour as an addiction is not a neutral move. It opens access to a structured framework of care and enables the engagement in treatment of patients who had hitherto experienced a degree of diagnostic and therapeutic wandering – a particularly salient issue given that help-seeking remains low in these disorders, with only around 10% of individuals with gambling disorder ever accessing treatment [43]. It also exposes them to a measurable – though lower than substance-related – public stigma documented as a barrier to help-seeking [44], and to the risk of overpathologisation of behavioural patterns that may reflect a more complex underlying condition rather than a primary disorder [37].

Finally, although we aimed to maximise transparency and reproducibility, some degree of subjectivity remains inherent to the framework. Reproducibility was strengthened through a PRISMA-based methodology, predefined eligibility criteria, and independent validation by two research teams from different countries. Nevertheless, certain steps still relied partly on expert judgment.

Using AUD as an external reference for a well-characterised substance-related addiction and gambling disorder as the gold standard for behavioural addictions, our synthesis allows us to propose the following provisional positions: (1) continued support for gambling and gaming disorder as an addictive disorder; (2) compelling, though not definitive, evidence that BED and CSBD share several core features with gambling disorder and merit serious consideration as behavioural addictions in future nosology, pending longitudinal and multi-domain confirmation; and (3) compulsive buying–shopping disorder, exercise addiction and work addiction require more rigorous, harmonised, multimodal studies before reclassification is justified, although the limited available data indicate congruence between these and gambling disorder. Much of the apparent heterogeneity likely reflects uneven empirical coverage and definitional inconsistencies. Rather than prematurely expanding or contracting the addiction category, the field needs coordinated, high-quality, longitudinal, multimodal research to determine which behavioural syndromes genuinely share the clinical, cognitive, neurobiological, and genetic architecture of substance use disorders.

## Supporting information

10.1192/j.eurpsy.2026.12249.sm001 Vansteene et al. supplementary material Vansteene et al. supplementary material

## Data Availability

Data sharing is not applicable – no new data generated.

## Supplementary material

The supplementary material for this article can be found at http://doi.org/10.1192/j.eurpsy.2026.12249.

## Long descriptions

### Figure 1. Long description

The flowchart follows a vertical progression through four main stages.

1. Identification: At the top, a box indicates 7606 references retrieved, broken down by database: 1201 in PubMed, 1136 in PsycInfo, 2497 in Scopus, and 2772 in Web of Science. An arrow points down to the next stage, with a side branch to the right indicating 3009 duplicates removed.

2. Screening: The next central box shows 4597 titles or abstracts screened. An arrow points down, with a side branch to the right indicating 3861 records excluded.

3. Eligibility: The third central box shows 736 full-text articles screened. An arrow points down, with a side branch to the right detailing 490 articles excluded for specific reasons: 250 not about factors in the table, 117 with no results specific to the addiction, 102 not a systematic review, 23 not in English, and 1 article published before 2000.

4. Included: The final box at the bottom indicates 246 studies included.

Navigate back to Figure 1..

### Table 1. Long description

The table consists of seven columns representing different disorders: Alcohol use disorder, Gaming disorder, Binge eating disorder, Compulsive sexual behaviour disorder, Work addiction, Exercise addiction, and Compulsive buying–shopping disorder. All values are percentages.

* Data availability: Alcohol use disorder 100.0, Gaming disorder 69.3, Binge eating disorder 54.7, Compulsive sexual behaviour disorder 48.0, Work addiction 20.0, Exercise addiction 20.0, Compulsive buying–shopping disorder 22.7.

* Unweighted concordance:

- Congruent: Alcohol use disorder 92.2, Gaming disorder 98.9, Binge eating disorder 84.9, Compulsive sexual behaviour disorder 89.7, Work addiction 70.7, Exercise addiction 66.7, Compulsive buying–shopping disorder 73.8.

- Incongruent: Alcohol use disorder 0.0, Gaming disorder 0.0, Binge eating disorder 4.0, Compulsive sexual behaviour disorder 0.0, Work addiction 3.3, Exercise addiction 4.8, Compulsive buying–shopping disorder 6.3.

- Inconclusive: Alcohol use disorder 7.8, Gaming disorder 1.1, Binge eating disorder 11.1, Compulsive sexual behaviour disorder 10.3, Work addiction 26.0, Exercise addiction 28.6, Compulsive buying–shopping disorder 20.0.

* Weighted concordance:

- Congruent: Alcohol use disorder 119.3, Gaming disorder 85.2, Binge eating disorder 75.2, Compulsive sexual behaviour disorder 50.1, Work addiction 84.5, Exercise addiction 60.2, Compulsive buying–shopping disorder 45.2.

- Incongruent: Alcohol use disorder 0.0, Gaming disorder 0.0, Binge eating disorder 1.9, Compulsive sexual behaviour disorder 0.0, Work addiction 3.3, Exercise addiction 3.9, Compulsive buying–shopping disorder 6.3.

- Inconclusive: Alcohol use disorder 9.9, Gaming disorder 0.7, Binge eating disorder 7.7, Compulsive sexual behaviour disorder 4.9, Work addiction 24.5, Exercise addiction 22.9, Compulsive buying–shopping disorder 14.1.

Navigate back to Table 1..

### Figure 2. Long description

The grid consists of seven rows, each representing a disorder: Alcohol use disorder, Gaming disorder, Binge eating disorder, Compulsive sexual behaviour disorder, Work addiction, Exercise addiction, and Compulsive buying-shopping disorder. Each row contains three panels.

Panel A: Data availability. Horizontal blue bars show the percentage of available data (0 to 100 percent) for nine categories: Global environment, Psychiatric comorbidity, Addictive comorbidity, Somatic comorbidity, Personality and temperament, Cognitive characteristics, Emotional dysregulation, Genetics, and Brain assessments. Alcohol use disorder shows 100 percent availability across all categories, while others vary significantly, with Work addiction and Exercise addiction showing the lowest overall availability.

Panel B: Unweighted concordance. Horizontal bars use color coding: green for congruent data, red for incongruent data, and grey for inconclusive data. Alcohol use disorder is predominantly green with some grey in Somatic comorbidity and Personality and temperament. Other disorders show varying levels of green, with notable red incongruent segments in Binge eating disorder (Global environment and Brain assessments), Work addiction (Personality and temperament), and Exercise addiction (Emotional dysregulation).

Panel C: Weighted concordance. Similar to Panel B but weighted by evidence level, with the x-axis extending up to 170 percent for some disorders. Alcohol use disorder exceeds 100 percent in several categories like Psychiatric comorbidity and Emotional dysregulation. The distribution of green, red, and grey segments shifts based on the weighting, often expanding the inconclusive grey areas or emphasizing the congruent green findings.

Navigate back to Figure 2..
