# Supplementary material for: Concordance between gambling disorder, alcohol use disorder, and other behavioural addictions provides evidence for a comprehensive approach to addictive behaviours: A multiple systematic review analysis
Source: Eur Psychiatry. 2026 Aug 12;69(1):e94. doi: 10.1192/j.eurpsy.2026.12249 (PMC13735838; doi:10.1192/j.eurpsy.2026.12249)
Supplement: Supplementary file 1 [file S0924933826122494sup001.docx]

Supplementary materials

**Supplementary Table S1.** PRISMA guidelines checklist.

|  | | | |
| --- | --- | --- | --- |
| **Section and Topic** | **Item #** | **Checklist item** | **Location where item is reported** |
| **TITLE** | | |  |
| Title | 1 | Identify the report as a systematic review. | Title |
| **ABSTRACT** | | |  |
| Abstract | 2 | See the PRISMA 2020 for Abstracts checklist. | Abstract |
| **INTRODUCTION** | | |  |
| Rationale | 3 | Describe the rationale for the review in the context of existing knowledge. | Intro p.3 |
| Objectives | 4 | Provide an explicit statement of the objective(s) or question(s) the review addresses. | Intro p.4 |
| **METHODS** | | |  |
| Eligibility criteria | 5 | Specify the inclusion and exclusion criteria for the review and how studies were grouped for the syntheses. | Search strategy p.6 |
| Information sources | 6 | Specify all databases, registers, websites, organisations, reference lists and other sources searched or consulted to identify studies. Specify the date when each source was last searched or consulted. | Search strategy p.6 |
| Search strategy | 7 | Present the full search strategies for all databases, registers and websites, including any filters and limits used. | Search strategy p.6 |
| Selection process | 8 | Specify the methods used to decide whether a study met the inclusion criteria of the review, including how many reviewers screened each record and each report retrieved, whether they worked independently, and if applicable, details of automation tools used in the process. | Study selection p.6 |
| Data collection process | 9 | Specify the methods used to collect data from reports, including how many reviewers collected data from each report, whether they worked independently, any processes for obtaining or confirming data from study investigators, and if applicable, details of automation tools used in the process. | Data extraction p.7 |
| Data items | 10a | List and define all outcomes for which data were sought. Specify whether all results that were compatible with each outcome domain in each study were sought (e.g. for all measures, time points, analyses), and if not, the methods used to decide which results to collect. | Data extraction p.7, Data synthesis p.8 |
|  | 10b | List and define all other variables for which data were sought (e.g. participant and intervention characteristics, funding sources). Describe any assumptions made about any missing or unclear information. | n/a |
| Study risk of bias assessment | 11 | Specify the methods used to assess risk of bias in the included studies, including details of the tool(s) used, how many reviewers assessed each study and whether they worked independently, and if applicable, details of automation tools used in the process. | Level of evidence assessment p.6 |
| Effect measures | 12 | Specify for each outcome the effect measure(s) (e.g. risk ratio, mean difference) used in the synthesis or presentation of results. | Data synthesis p.8 |
| Synthesis methods | 13a | Describe the processes used to decide which studies were eligible for each synthesis (e.g. tabulating the study intervention characteristics and comparing against the planned groups for each synthesis (item #5)). | Data synthesis p.8 |
|  | 13b | Describe any methods required to prepare the data for presentation or synthesis, such as handling of missing summary statistics, or data conversions. | Data synthesis p.8 |
|  | 13c | Describe any methods used to tabulate or visually display results of individual studies and syntheses. | Data synthesis p.8 |
|  | 13d | Describe any methods used to synthesize results and provide a rationale for the choice(s). If meta-analysis was performed, describe the model(s), method(s) to identify the presence and extent of statistical heterogeneity, and software package(s) used. | Data synthesis p.8 |
|  | 13e | Describe any methods used to explore possible causes of heterogeneity among study results (e.g. subgroup analysis, meta-regression). | Level of evidence assessment p.6 |
|  | 13f | Describe any sensitivity analyses conducted to assess robustness of the synthesized results. | n/a |
| Reporting bias assessment | 14 | Describe any methods used to assess risk of bias due to missing results in a synthesis (arising from reporting biases). | n/a |
| Certainty assessment | 15 | Describe any methods used to assess certainty (or confidence) in the body of evidence for an outcome. | n/a |
| **RESULTS** | | |  |
| Study selection | 16a | Describe the results of the search and selection process, from the number of records identified in the search to the number of studies included in the review, ideally using a flow diagram. | Figure S1, Study characteristics p.9 |
|  | 16b | Cite studies that might appear to meet the inclusion criteria, but which were excluded, and explain why they were excluded. | Data synthesis p.8 |
| Study characteristics | 17 | Cite each included study and present its characteristics. | Table S4 |
| Risk of bias in studies | 18 | Present assessments of risk of bias for each included study. | Table S4 |
| Results of individual studies | 19 | For all outcomes, present, for each study: (a) summary statistics for each group (where appropriate) and (b) an effect estimate and its precision (e.g. confidence/credible interval), ideally using structured tables or plots. | n/a |
| Results of syntheses | 20a | For each synthesis, briefly summarise the characteristics and risk of bias among contributing studies. | Study characteristics p.9 |
|  | 20b | Present results of all statistical syntheses conducted. If meta-analysis was done, present for each the summary estimate and its precision (e.g. confidence/credible interval) and measures of statistical heterogeneity. If comparing groups, describe the direction of the effect. | Data availability p.10, Unweighted concordance p.10, Weighted concordance p.11 |
|  | 20c | Present results of all investigations of possible causes of heterogeneity among study results. | n/a |
|  | 20d | Present results of all sensitivity analyses conducted to assess the robustness of the synthesized results. | n/a |
| Reporting biases | 21 | Present assessments of risk of bias due to missing results (arising from reporting biases) for each synthesis assessed. | Weighted concordance p.11 |
| Certainty of evidence | 22 | Present assessments of certainty (or confidence) in the body of evidence for each outcome assessed. | n/a |
| **DISCUSSION** | | |  |
| Discussion | 23a | Provide a general interpretation of the results in the context of other evidence. | Discussion p.12 |
|  | 23b | Discuss any limitations of the evidence included in the review. | Discussion p.13 |
|  | 23c | Discuss any limitations of the review processes used. | Discussion p.13 |
|  | 23d | Discuss implications of the results for practice, policy, and future research. | Discussion p.15 |
| **OTHER INFORMATION** | | |  |
| Registration and protocol | 24a | Provide registration information for the review, including register name and registration number, or state that the review was not registered. | n/a |
|  | 24b | Indicate where the review protocol can be accessed, or state that a protocol was not prepared. | n/a |
|  | 24c | Describe and explain any amendments to information provided at registration or in the protocol. | n/a |
| Support | 25 | Describe sources of financial or non-financial support for the review, and the role of the funders or sponsors in the review. | Financial support p.16 |
| Competing interests | 26 | Declare any competing interests of review authors. | Conflicts of interest p.16 |
| Availability of data, code and other materials | 27 | Report which of the following are publicly available and where they can be found: template data collection forms; data extracted from included studies; data used for all analyses; analytic code; any other materials used in the review. | Data availability statement p.16 |

**Supplementary Table S2.** Search strings used in each database for each addiction

| **Gambling disorder** |  |
| --- | --- |
| PubMed | (gambl*[Title/Abstract]) |
| PsycInfo | TI (gambl*) OR AB (gambl*) |
| Scopus | ( TITLE-ABS-KEY ( gambl*  AND [systematic review OR meta-analysis] )  AND  PUBYEAR  >  1999 |
| Web of Science | (gambl*) (Topic) and systematic review OR meta-analysis (All Fields) |
| **Gaming disorder** |  |
| PubMed | (videogaming addiction[Title/Abstract]) OR (videogaming disorder[Title/Abstract]) OR (pathological videogaming[Title/Abstract]) OR (gaming addiction[Title/Abstract]) OR (gaming disorder[Title/Abstract]) OR (pathological gaming[Title/Abstract]) |
| PsycInfo | TI ( (videogaming addiction) OR (videogaming disorder) OR (pathological videogaming) OR (gaming addiction) OR (gaming disorder) OR (pathological gaming) ) OR AB ( (videogaming addiction) OR (videogaming disorder) OR (pathological videogaming) OR (gaming addiction) OR (gaming disorder) OR (pathological gaming) ) |
| Scopus | ( TITLE-ABS-KEY ( ( "videogaming addiction" )  OR  ( "videogaming disorder" )  OR  ( "pathological videogaming" )  OR  ( "gaming addiction" )  OR  ( "gaming disorder" )  OR  ( "pathological gaming" ) ) AND  TITLE-ABS-KEY ( ( systematic  AND  review )  OR  ( meta-analysis ) ) )  AND PUBYEAR > 1999 AND  PUBYEAR  >  1999 |
| Web of Science | (videogaming addiction) OR (videogaming disorder) OR (pathological videogaming) OR (gaming addiction) OR (gaming disorder) OR (pathological gaming) (Topic) and systematic review OR meta-analysis (All Fields) |
| **Binge eating disorder** | |
| PubMed | (binge-eating[Title/Abstract]) OR (compulsive overeating[Title/Abstract]) OR (compulsive eating[Title/Abstract]) OR (food addiction[Title/Abstract]) |
| PsycInfo | TI ( (binge-eating) OR (compulsive overeating) OR (compulsive eating) OR (food addiction) ) OR AB ( (binge-eating) OR (compulsive overeating) OR (compulsive eating) OR (food addiction) ) |
| Scopus | ( TITLE-ABS-KEY ( ( binge-eating )  OR  ( "compulsive overeating" )  OR  ( "compulsive eating" ) OR ( "food addiction" ) )  AND  TITLE-ABS-KEY ( ( systematic  AND  review )  OR  ( meta-analysis ) ) ) AND PUBYEAR  >  1999  AND  PUBYEAR  >  1999 |
| Web of Science | (binge-eating) OR (compulsive overeating) OR (compulsive eating) OR (food addiction) (Topic) and systematic review OR meta-analysis (All Fields) |
| **Compulsive sexual behaviour disorder** | |
| PubMed | (sex addiction[Title/Abstract]) OR (sex addict[Title/Abstract]) OR (sexual addiction[Title/Abstract]) OR (hypersexual*[Title/Abstract]) OR (compulsive sexual[Title/Abstract]) |
| PsycInfo | TI ( (sex addiction) OR (sex addict) OR (sexual addiction) OR (hypersexual*) OR (compulsive sexual) ) OR AB ( (sex addiction) OR (sex addict) OR (sexual addiction) OR (hypersexual*) OR (compulsive sexual) ) |
| Scopus | ( TITLE-ABS-KEY ( ( "sex addiction" )  OR  ( "sex addict" )  OR  ( "sexual addiction" ) OR  ( hypersexual* ) OR  ( "compulsive sexual" ) )  AND  TITLE-ABS-KEY ( ( systematic AND review ) OR  ( meta-analysis ) ) ) AND  PUBYEAR  >  1999  AND  PUBYEAR  >  1999 |
| Web of Science | (sex addiction) OR (sex addict) OR (sexual addiction) OR (hypersexual*) OR (compulsive sexual) (Topic) and systematic review OR meta-analysis (All Fields) |
| **Work addiction** |  |
| PubMed | (work addiction[Title/Abstract]) OR (compulsive working[Title/Abstract]) OR (workaholism[Title/Abstract]) |
| PsycInfo | TI ( (work addiction) OR (compulsive working) OR (workaholism) ) OR AB ( (work addiction) OR (compulsive working) OR (workaholism) ) |
| Scopus | ( TITLE-ABS-KEY ( ( "work addiction" )  OR  ( "compulsive work" )  OR  ( workaholism ) )  AND  TITLE-ABS-KEY ( ( systematic  AND  review )  OR  ( meta-analysis ) ) )  AND PUBYEAR  >  1999  AND PUBYEAR >  1999 |
| Web of Science | (work addiction) OR (compulsive work) OR (workaholism) (Topic) and systematic review OR meta-analysis (All Fields) |
| **Exercise addiction** | |
| PubMed | (exercise addiction[Title/Abstract]) OR (exercise dependence[Title/Abstract]) OR (excessive exercise[Title/Abstract]) OR (compulsive exercise[Title/Abstract]) |
| PsycInfo | TI ( (exercise addiction) OR (exercise dependence) OR (excessive exercise) OR (compulsive exercise) ) OR AB ( (exercise addiction) OR (exercise dependence) OR (excessive exercise) OR (compulsive exercise) ) |
| Scopus | ( TITLE-ABS-KEY ( ( "exercise addiction" )  OR  ( "exercise dependence" )  OR  ( "excessive exercise" ) OR ( "compulsive exercise" ) )  AND  TITLE-ABS-KEY ( ( systematic  AND  review )  OR  ( meta-analysis ) ) ) AND  PUBYEAR  >  1999  AND  PUBYEAR  >  1999 |
| Web of Science | (exercise addiction) OR (exercise dependence) OR (excessive exercise) OR (compulsive exercise) (Topic) and systematic review OR meta-analysis (All Fields) |
| **Compulsive buying-shopping disorder** | |
| PubMed | (compulsive buying[Title/Abstract]) OR (pathological buying[Title/Abstract]) OR (buying disorder[Title/Abstract]) OR (buying addiction[Title/Abstract]) OR (shopping addiction[Title/Abstract]) OR (shopping disorder[Title/Abstract]) OR (compulsive shopping[Title/Abstract]) OR (pathological shopping[Title/Abstract]) |
| PsycInfo | TI ( (compulsive buying) OR (pathological buying) OR (buying disorder) OR (buying addiction) OR (shopping addiction) OR (shopping disorder) OR (compulsive shopping) OR (pathological shopping) ) OR AB ( (compulsive buying) OR (pathological buying) OR (buying disorder) OR (buying addiction) OR (shopping addiction) OR (shopping disorder) OR (compulsive shopping) OR (pathological shopping) ) |
| Scopus | ( TITLE-ABS-KEY ( ( "compulsive buying" )  OR  ( "pathological buying" )  OR  ( "buying disorder" )  OR ( "buying addiction" )  OR  ( "shopping addiction" )  OR  ( "shopping disorder" )  OR  ( "compulsive shopping" ) OR  ( "pathological shopping" ) )  AND  TITLE-ABS-KEY ( ( systematic  AND  review ) OR ( meta-analysis ) ) )  AND  PUBYEAR  >  1999  AND  PUBYEAR  >  1999 |
| Web of Science | (compulsive buying) OR (pathological buying) OR (buying disorder) OR (buying addiction) OR (shopping addiction) OR (shopping disorder) OR (compulsive shopping) OR (pathological shopping) (Topic) and systematic review OR meta-analysis (All Fields) |

*Note: The literature search included searching for meta-analyses or systematic reviews from 2000 onwards containing the name of the behavioural addiction in the title or the abstract.*

*On PubMed and PsycInfo, filters concerning the type of study (“meta-analysis” and “systematic review”) and the year of publication were manually selected. On Web of Science, the type of study was included in the search string, but the filter concerning the year of publication was manually selected. On Scopus, both filters were included in the search string.*

**Supplementary Figure S1.** PRISMA flow charts for each individual behavioural addiction. A. Gambling disorder, B. Gaming disorder, C. Binge eating disorder, D. Compulsive sexual behaviour disorder, E. Work addiction, F. Exercise addiction, G. Compulsive shopping-buying addiction.

| **Table S3.** Detailed list of factors and their associations with each disorder. | | | | | | | | | | | | | | | | | | |
| --- | --- | --- | --- | --- | --- | --- | --- | --- | --- | --- | --- | --- | --- | --- | --- | --- | --- | --- |
| **Factors** | | | **GbD** | | **AUD** | | **GmD** | | **BED** | | **CSBD** | | **WA** | | **EA** | | **CBSD** | |
|  |  |  | **Association** | **LoE** | **Association** | **LoE** | **Association** | **LoE** | **Association** | **LoE** | **Association** | **LoE** | **Association** | **LoE** | **Association** | **LoE** | **Association** | **LoE** |
| **1-Global environment** | | |  |  |  |  |  |  |  |  |  |  |  |  |  |  |  |  |
|  | General characteristics | |  |  |  |  |  |  |  |  |  |  |  |  |  |  |  |  |
|  |  | Gender (males) | Positive | 1++ | Positive | 1+++ | Positive | 1++ | Negative | 1+ | Positive | 2+ | Inconclusive | 1+ | Positive | 1++ | Negative | 1++ |
|  |  | Age (younger than 30) | Positive | 1++ | Positive | 1+++ | Positive | 1++ | Positive | 2++ | Positive | 2+ | Inconclusive | 1+ | Positive | 1++ | Positive | 1++ |
|  |  | Ethnicity (minorities) | Positive | 1++ | Inconclusive | 1+++ | Positive | 2+ | Inconclusive | 2++ |  |  |  |  |  |  |  |  |
|  | Situation | |  |  |  |  |  |  |  |  |  |  |  |  |  |  |  |  |
|  |  | Education (low) | Positive | 1++ | Inconclusive | 1+++ | Positive | 1++ | Inconclusive | 2++ |  |  | Inconclusive | 1+ |  |  |  |  |
|  |  | Socio-economic status (low) | Positive | 1++ | Positive | 1+++ | Positive | 2+ | Inconclusive | 2++ |  |  |  |  |  |  |  |  |
|  |  | Social support (low) | Positive | 2+ | Inconclusive | 1+++ | Positive | 1++ |  |  |  |  |  |  | Positive | 2++ |  |  |
|  |  | Social functioning (low) | Positive | 2+ | Positive | 1+++ |  |  | Positive | 2- |  |  | Positive | 1++ |  |  |  |  |
|  | Familial | |  |  |  |  |  |  |  |  |  |  |  |  |  |  |  |  |
|  |  | Marital status (single or separated) | Positive | 1++ | Positive | 1+++ | Positive | 2+ |  |  | Inconclusive | 2+ | Inconclusive | 1+ |  |  |  |  |
|  |  | Not living with both parents | Positive | 2+ | Positive | 1+ | Positive | 1++ |  |  |  |  |  |  |  |  |  |  |
|  |  | Parent supervision (less) | Positive | 1++ | Positive | 1++ | Positive | 1++ |  |  |  |  |  |  |  |  |  |  |
|  | Trauma, stress and criminality | |  |  |  |  |  |  |  |  |  |  |  |  |  |  |  |  |
|  |  | Stressful life events | Positive | 2+ | Positive | 2- | Positive | 1++ | Positive | 2++ | Positive | 2+ |  |  |  |  |  |  |
|  |  | History of physical, sexual or emotional abuse | Positive | 2+ | Positive | 1++ | Positive | 1++ | Positive | 1++ | Positive | 2+ |  |  |  |  |  |  |
|  |  | Peer antisocial behaviours | Positive | 1++ | Positive | 2+ |  |  |  |  |  |  |  |  |  |  |  |  |
|  |  | Domestic violence victimisation | Positive | 2+ | Positive | 1++ |  |  | Positive | 2+ |  |  |  |  |  |  |  |  |
|  |  | Perpetration of violence on partner or children | Positive | 1++ | Positive | 1+ |  |  |  |  |  |  |  |  |  |  |  |  |
|  |  | Delinquency and/or criminal activities | Positive | 1++ | Positive | 1+++ |  |  |  |  |  |  |  |  |  |  |  |  |
| **2-Psychiatric comorbidity** | | |  |  |  |  |  |  |  |  |  |  |  |  |  |  |  |  |
|  | Global & suicidal risk | |  |  |  |  |  |  |  |  |  |  |  |  |  |  |  |  |
|  |  | Any mental health disorder | Positive | 1++ | Positive | 1+++ | Positive | 1++ | Positive | 2- | Positive | 2+ |  |  | Positive | 1++ |  |  |
|  |  | Low level of perceived health | Positive | 2+ | Positive | 1+++ |  |  |  |  |  |  |  |  |  |  |  |  |
|  |  | Suicidal ideation (presence) | Positive | 1++ | Positive | 1++ | Positive | 1++ | Positive | 2++ |  |  |  |  |  |  |  |  |
|  |  | Attempted suicide (lifetime) | Positive | 1++ | Positive | 1++ | Positive | 2++ | Positive | 1++ | Positive | 2+ |  |  |  |  |  |  |
|  | Disorders | |  |  |  |  |  |  |  |  |  |  |  |  |  |  |  |  |
|  |  | Anxiety disorder | Positive | 1++ | Positive | 1+++ | Positive | 1++ | Positive | 2++ | Positive | 2+ | Positive | 1++ | Positive | 1++ | Positive | 2+ |
|  |  | PTSD | Positive | 2+ | Positive | 1+++ |  |  | Positive | 2+ | Positive | 2+ |  |  |  |  |  |  |
|  |  | Bipolar disorder | Positive | 2+ | Positive | 1+++ |  |  | Positive | 2++ | Positive | 1+ |  |  |  |  | Positive | 2+ |
|  |  | Depression | Positive | 1++ | Positive | 1+++ | Positive | 1++ | Positive | 2++ | Positive | 1++ |  |  | Positive | 1++ | Positive | 2- |
|  |  | ADHD (any) | Positive | 1+ | Positive | 1+++ | Positive | 1++ | Positive | 1++ | Positive | 1++ |  |  |  |  |  |  |
|  |  | Childhood ADHD | Positive | 2+ | Positive | 1++ |  |  |  |  |  |  |  |  |  |  |  |  |
|  |  | Conduct disorder | Positive | 2+ | Positive | 1+++ | Positive | 2+ |  |  |  |  |  |  |  |  |  |  |
|  |  | Learning disorders | Positive | 2+ | Positive | 2+ | Positive | 2+ |  |  | Positive | 2+ |  |  |  |  |  |  |
| **3-Addictive comorbidity** | | |  |  |  |  |  |  |  |  |  |  |  |  |  |  |  |  |
|  | Substance use and abuse | |  |  |  |  |  |  |  |  |  |  |  |  |  |  |  |  |
|  |  | Any substance use disorder | Positive | 2++ | Positive | 1+++ | Positive | 2+ | Positive | 2- | Positive | 2+ |  |  |  |  | Positive | 2+ |
|  |  | Alcohol | Positive | 1++ | NA | NA | Positive | 1+ | Positive | 1++ | Positive | 2+ |  |  | Inconclusive | 2++ |  |  |
|  |  | Tobacco/nicotine | Positive | 1++ | Positive | 1+++ |  |  | Positive | 1++ | Positive | 2+ |  |  | Inconclusive | 2++ |  |  |
|  |  | Illicit drug | Positive | 1++ | Positive | 1+++ |  |  | Positive | 1++ | Positive | 2+ |  |  |  |  |  |  |
|  |  | Cannabis | Positive | 1++ | Positive | 1+++ |  |  |  |  |  |  |  |  |  |  |  |  |
|  |  | Cocaine | Positive | 1++ | Positive | 1+++ |  |  |  |  |  |  |  |  |  |  |  |  |
|  |  | Marijuana | Positive | 1++ | Positive | 1+++ |  |  |  |  |  |  |  |  |  |  |  |  |
| **4-Somatic comorbidity** | | |  |  |  |  |  |  |  |  |  |  |  |  |  |  |  |  |
|  |  | Poor physical health and chronic medical conditions | Positive | 1++ | Positive | 1++ | Positive | 2+ | Positive | 2++ | Positive | 2+ |  |  | Inconclusive | 1++ |  |  |
|  |  | Traumatic brain injury | Positive | 2+ | Positive | 1++ |  |  |  |  | Positive | 2+ |  |  |  |  |  |  |
|  |  | Parkinson's disease | Positive | 1++ | Inconclusive | 1++ |  |  | Positive | 1++ | Positive | 1++ |  |  |  |  | Inconclusive | 1++ |
| **5-Personality and temperament** | | |  |  |  |  |  |  |  |  |  |  |  |  |  |  |  |  |
|  | Temperament | |  |  |  |  |  |  |  |  |  |  |  |  |  |  |  |  |
|  |  | Antisocial behaviours | Positive | 1++ | Positive | 1+++ | Positive | 2+ |  |  |  |  |  |  |  |  |  |  |
|  |  | Obsessive-compulsive traits | Positive | 1++ | Inconclusive | 1+ |  |  | Positive | 1++ | Positive | 2+ | Positive | 1++ | Positive | 1++ |  |  |
|  | Personality disorder | |  |  |  |  |  |  |  |  |  |  |  |  |  |  |  |  |
|  |  | Antisocial personality disorder | Positive | 2+ | Positive | 1+++ |  |  |  |  |  |  |  |  |  |  |  |  |
|  | Personality traits (assessed through tests) | |  |  |  |  |  |  |  |  |  |  |  |  |  |  |  |  |
|  |  | Agreeableness (less) | Positive | 1++ | Positive | 1++ | Positive | 1++ |  |  | Inconclusive | 2+ | Inconclusive | 1++ |  |  |  |  |
|  |  | Conscientiousness (less) | Positive | 1++ | Positive | 1++ | Positive | 1++ |  |  | Inconclusive | 2+ | Positive | 1++ |  |  |  |  |
|  |  | Extraversion (less) | Positive | 1++ | Positive | 1++ | Positive | 1++ |  |  | Inconclusive | 2+ | Negative | 1++ |  |  |  |  |
|  |  | Neuroticism (more) | Positive | 1++ | Inconclusive | 1++ | Positive | 1++ | Positive | 2- | Positive | 2+ | Inconclusive | 1++ |  |  | Positive | 2+ |
|  |  | Openness to experience (less) | Positive | 1++ | Positive | 1++ | Positive | 1++ |  |  | Inconclusive | 2+ | Inconclusive | 1++ |  |  |  |  |
|  |  | Disinhibition | Positive | 1+ | Positive | 1++ | Positive | 1++ |  |  |  |  |  |  |  |  |  |  |
|  |  | Low premeditation | Positive | 1+ | Positive | 1++ | Positive | 1++ |  |  | Inconclusive | 2++ |  |  |  |  |  |  |
|  |  | Negative urgency | Positive | 1+ | Positive | 1++ | Positive | 1++ | Inconclusive | 2- | Positive | 2++ |  |  |  |  |  |  |
| **6-Cognitive specificities** | | |  |  |  |  |  |  |  |  |  |  |  |  |  |  |  |  |
|  | Impulsivity and decision-making | |  |  |  |  |  |  |  |  |  |  |  |  |  |  |  |  |
|  |  | Impulsivity (in general) | Positive | 1++ | Positive | 1++ | Positive | 1++ |  |  | Positive | 2++ | Positive | 1++ |  |  |  |  |
|  |  | Attentional inhibition impairments (Stroop) | Positive | 1++ | Positive | 1++ | Positive | 1++ | Inconclusive | 1++ |  |  |  |  |  |  |  |  |
|  |  | Response inhibition (GNG, SST...) | Positive | 1+ | Positive | 1++ | Positive | 1++ | Positive | 1++ |  |  |  |  |  |  | Inconclusive | 2- |
|  |  | Decision-making impairments (IGT) | Positive | 1++ | Positive | 1++ | Positive | 1++ | Positive | 1++ |  |  |  |  |  |  | Positive | 2- |
|  |  | Delay discounting (greater preference for smaller-immediate rewards over larger-delayed rewards) | Positive | 1++ | Positive | 1++ | Positive | 1++ | Positive | 1++ |  |  |  |  |  |  | Positive | 2++ |
|  |  | Probability discounting (risky decision-making) | Positive | 1++ | Positive | 1++ | Positive | 1++ |  |  |  |  |  |  |  |  |  |  |
|  | Other cognition and psychological processes | |  |  |  |  |  |  |  |  |  |  |  |  |  |  |  |  |
|  |  | Cognitive bias/distorted cognitions | Positive | 2++ | Positive | 1++ |  |  |  |  |  |  |  |  |  |  |  |  |
|  |  | Cognitive flexibility (lack of) | Positive | 1++ | Positive | 1++ |  |  | Positive | 1++ | Positive | 2+ |  |  |  |  | Inconclusive | 2- |
|  |  | Attentional bias/task set-shifting | Positive | 1++ | Positive | 1++ | Inconclusive | 2++ | Inconclusive | 1++ |  |  |  |  |  |  | Inconclusive | 2- |
|  |  | Beliefs/superstitions | Positive | 2++ | Positive | 1++ |  |  |  |  |  |  |  |  |  |  |  |  |
|  |  | Dissociation | Positive | 1++ | Positive | 1++ | Positive | 2+ |  |  | Positive | 2+ |  |  |  |  |  |  |
|  |  | Internalizing symptoms | Positive | 1++ | Positive | 1++ |  |  |  |  |  |  |  |  |  |  |  |  |
|  |  | Metacognitive deficits | Positive | 1++ | Positive | 2+ | Positive | 2++ |  |  |  |  |  |  |  |  |  |  |
|  |  | Probability discounting | Positive | 1++ | Positive | 1++ | Positive | 1++ |  |  |  |  |  |  |  |  |  |  |
| **7-Emotional dysregulation** | | |  |  |  |  |  |  |  |  |  |  |  |  |  |  |  |  |
|  |  | Alexithymia | Positive | 2+ | Positive | 1++ | Positive | 2+ | Positive | 1++ |  |  |  |  |  |  |  |  |
|  |  | Emotion regulation deficit | Positive | 1++ | Positive | 1++ | Positive | 1++ | Positive | 1++ | Positive | 2+ |  |  | Positive | 1++ |  |  |
|  |  | Emotion regulation strategy: more suppression | Positive | 1++ | Positive | 1++ | Positive | 2+ | Positive | 1++ |  |  |  |  |  |  |  |  |
|  |  | Emotional distress | Positive | 2+ | Positive | 1+++ | Positive | 1+ |  |  | Positive | 2+ |  |  |  |  |  |  |
|  |  | Negative affects | Positive | 1+ | Positive | 1++ | Positive | 2+ |  |  | Positive | 2+ | Positive | 1++ | Positive | 2++ |  |  |
|  |  | Low self-esteem | Positive | 2+ | Positive | 1+++ | Positive | 1++ | Positive | 1++ | Positive | 2- | Positive | 1++ | Negative | 2++ | Positive | 2+ |
| **8-Genetics** | | |  |  |  |  |  |  |  |  |  |  |  |  |  |  |  |  |
|  |  | Contribution of genetic factors (twin studies) | Positive | 1+ | Positive | 1+++ |  |  |  |  |  |  |  |  |  |  |  |  |
|  |  | Associated alleles of dopamine gene(s) | Positive | 2++ | Positive | 1+ | Positive | 2+ | Positive | 2++ |  |  |  |  |  |  |  |  |
| **9-Brain assessments** | | |  |  |  |  |  |  |  |  |  |  |  |  |  |  |  |  |
|  |  | Peripheral response to addiction-related cues | Positive | 1++ | Positive | 1++ |  |  |  |  | Positive | 2+ |  |  |  |  | Positive | 1++ |
|  |  | EEG abnormalities | Positive | 1++ | Positive | 1++ | Positive | 1++ | Positive | 2++ | Positive | 2+ |  |  |  |  |  |  |
|  | **Brain anatomy** | |  |  |  |  |  |  |  |  |  |  |  |  |  |  |  |  |
|  | Grey matter alterations | | Positive | 1++ | Positive | 1++ |  |  |  |  | Positive | 2+ |  |  |  |  |  |  |
|  |  | *L medial superior frontal gyrus GM reduction* | Positive | 1++ | Positive | 1++ | Positive | 1++ |  |  |  |  |  |  |  |  |  |  |
|  |  | *R medial superior frontal gyrus GM reduction* | Positive | 1++ | Inconclusive | 1++ | Positive | 1++ |  |  |  |  |  |  |  |  |  |  |
|  |  | *R medial orbitofrontal gyrus GM reduction* | Positive | 1++ | Inconclusive | 1++ | Positive | 2+ | Negative | 2- |  |  |  |  | Positive | 2+ |  |  |
|  |  | *L medial orbitofrontal gyrus GM reduction* | Positive | 1++ | Inconclusive | 1++ | Positive | 2+ | Negative | 2- |  |  |  |  |  |  |  |  |
|  |  | *L anterior cingulate GM reduction* | Positive | 1++ | Positive | 1++ | Positive | 1++ | Negative | 2- |  |  |  |  |  |  |  |  |
|  |  | *R ventrolateral and ventromedial prefrontal cortex GM reduction* | Positive | 1++ | Positive | 1++ | Positive | 2- |  |  |  |  |  |  |  |  |  |  |
|  |  | *Cortical thinning hippocampus* | Positive | 2++ | Positive | 1++ | Positive | 1++ |  |  |  |  |  |  |  |  |  |  |
|  |  | *Cortical thickening right ventral striatum and prefrontal regions* | Positive | 2++ | Positive | 1++ | Positive | 1++ |  |  |  |  |  |  |  |  |  |  |
|  | White matter alterations | | Positive | 2++ | Positive | 1++ | Positive | 1++ |  |  | Positive | 2+ |  |  |  |  |  |  |
|  |  | *Microstructural damage in bilateral superior longitudinal fascicle, corpus callosum and corticospinal tract* | Positive | 2++ | Positive | 1++ | Positive | 1++ |  |  |  |  |  |  | Positive | 2+ |  |  |
|  | **Impaired brain activity (fMRI) at any level** | | Positive | 1++ | Positive | 1++ |  |  |  |  | Positive | 2+ |  |  |  |  |  |  |
|  |  | *Middle occipital gyrus: Hyperactivation (Response to addiction-related cues)* | Positive | 1+ | Inconclusive | 1+ |  |  | Positive | 2+ |  |  |  |  | Negative | 2+ |  |  |
|  |  | *Right inferior frontal gyrus: Hyperactivation (Response to addiction-related cues)* | Positive | 1+ | Inconclusive | 1+ | Positive | 1++ |  |  |  |  |  |  |  |  |  |  |
|  |  | *Right middle frontal gyrus: Hyperactivation (Decision-making)* | Positive | 1+ | Positive | 1++ | Positive | 1+ | Inconclusive | 2+ |  |  |  |  |  |  |  |  |
|  |  | *Right middle frontal gyrus: -correlation SOGS score (Response to addiction-related cues)* | Positive | 1+ | Positive | 1+ |  |  |  |  |  |  |  |  |  |  | Positive | 2- |
|  |  | *Medial prefrontal cortex: Hyperactivation (Gain processing)* | Positive | 1++ | Inconclusive | 1++ |  |  |  |  |  |  |  |  |  |  |  |  |
|  |  | *Bilateral dorsolateral prefrontal cortex: Hypoactivation (Decision-making+response inhibition (go/no-go))* | Positive | 2++ | Inconclusive | 2+ |  |  | Positive | 2+ |  |  |  |  |  |  | Positive | 2- |
|  |  | *Ventrolateral and medial prefrontal cortex: Hypoactivation (Loss processing)* | Positive | 2++ |  |  |  |  |  |  |  |  |  |  | Positive | 2+ |  |  |
|  |  | *Orbitofrontal cortex: Hyperactivation (Gain processing+decision making)* | Positive | 2++ | Positive | 1++ | Positive | 1++ | Positive | 2+ |  |  |  |  |  |  |  |  |
|  |  | *Median cingulate gyrus: Hyperactivation (Response to addiction-related cues)* | Positive | 1+ | Inconclusive | 1+ | Positive | 1++ |  |  |  |  |  |  | Positive | 2+ |  |  |
|  |  | *Anterior cingulate gyrus: Hyperactivation (Response to addiction-related cues)* | Positive | 2++ | Inconclusive | 1+ | Positive | 1+ | Positive | 2++ |  |  |  |  |  |  |  |  |
|  |  | *Left dorsal anterior cingulate cortex: Hypoactivation (Loss processing)* | Positive | 1++ |  |  |  |  |  |  |  |  |  |  |  |  |  |  |
|  |  | *Right striatum: Hyperactivation (Response to addiction-related cues)* | Positive | 1+ | Inconclusive | 1+ |  |  |  |  | Positive | 2+ |  |  |  |  | Positive | 2- |
|  |  | *Right ventral striatum: Hyperactivation (Decision-making + gain processing)* | Positive | 1++ | Inconclusive | 1++ |  |  |  |  |  |  |  |  |  |  |  |  |
|  |  | *Ventral striatum: Hypoactivation (Loss processing)* | Positive | 2++ | Inconclusive | 1++ |  |  | Positive | 2+ |  |  |  |  |  |  |  |  |
|  |  | *Bilateral striatum: Hypoactivation (Reward anticipation)* | Positive | 1+ | Inconclusive | 1++ |  |  | Positive | 2- |  |  |  |  |  |  |  |  |
|  |  | *Bilateral dorsal striatum: Hypoactivation (Reward outcome)* | Positive | 1+ | Inconclusive | 1++ |  |  |  |  |  |  |  |  |  |  |  |  |
|  |  | *Right lentiform nucleus: Hyperactivation+correlation SOGS score (Response to addiction-related cues)* | Positive | 1+ | Inconclusive | 1+ |  |  |  |  |  |  |  |  |  |  |  |  |
|  |  | *Right fusiform gyrus: Hyperactivation (Response to addiction-related cues)* | Positive | 1+ | Positive | 1+ | Positive | 1+ |  |  |  |  |  |  |  |  |  |  |
|  |  | *Caudate: Hyperactivation (Decision-making)* | Positive | 1+ | Positive | 1++ | Positive | 1++ |  |  |  |  |  |  |  |  |  |  |
|  |  | *Right putamen: Hyperactivation (Decision-making)* | Positive | 1+ | Inconclusive | 1++ | Positive | 2+ |  |  |  |  |  |  |  |  |  |  |
|  |  | *Bilateral para-hippocampus: +correlation SOGS score (Response to addiction-related cues)* | Positive | 1+ | Positive | 1+ | Positive | 2+ |  |  |  |  |  |  |  |  |  |  |
|  |  | *Hypothalamus: Hyperactivation (Decision-making)* | Positive | 1+ | Inconclusive | 1++ |  |  |  |  |  |  |  |  |  |  |  |  |
|  | **Dopamine dysregulation (PET & SPECT)** | | Positive | 2++ | Inconclusive | 2+ | Positive | 2- | Positive | 2+ |  |  |  |  |  |  |  |  |
|  |  | *Dopamine DRD2 function (PET): Same availability but greater release* | Positive | 2++ | Inconclusive | 2+ | Positive | 2- |  |  |  |  |  |  |  |  |  |  |
|  |  | *Dopamine transporter DAT function (SPECT): Lower availability* | Positive | 2++ |  |  |  |  |  |  |  |  |  |  |  |  |  |  |
| *AUD: alcohol use disorder, BED: binge eating disorder, CBSD: compulsive buying-shopping disorder, CSBD: compulsive sexual behaviour disorder, EA: exercise addiction, GbD: gambling disorder, GmD: gaming disorder, LoE: level of evidence, WA: work addiction* | | | | | | | | | | | | | | | | | | |

| **Table S4.** List of references included in the analysis. | | | |
| --- | --- | --- | --- |
| **Reference** | **LoE** | **Disorder** | **Factors studied** |
| Adolphe 2019 [1] | 2+ | GbD | Delinquency and/or criminal activities |
| Ágh 2015 [2] | 2++ | BED | Gender, Age, Ethnicity, Suicidal ideation, History of suicide attempt, Anxiety disorder, Bipolar disorder, Depression, Poor physical health and chronic medical conditions |
| Alcaraz-Ibáñez 2022 [3] | 1++ | EA | Gender |
| Alexandre 2019 [4] | 2+ | AUD | Dopamine DRD2 dysfunction (PET) |
| Allami 2021 [5] | 1++ | GbD | History of suicide attempt, Anxiety disorder, Age, Gender, Education, Ethnicity, Marital status, Delinquency and/or criminal activities, Any mental health disorder, Suicidal ideation, Depression, Alcohol, Tobacco/nicotine, Illicit drug, Cocaine, Marijuana, Poor physical health and chronic medical conditions, Internalizing symptoms |
| Álvarez Ruiz 2015 [6] | 2+ | BED | Bipolar disorder |
| Amendola 2025 [7] | 1++ | GmD | Depression, Anxiety |
| Amiri 2020 [8] | 1++ | AUD | History of suicide attempt |
| Amiri 2021 [9] | 1+ | AUD | Not living with both parents |
| Amiri 2025 [10] | 1++ | BED | History of physical, sexual or emotional abuse |
| Amlung 2017 [11] | 1++ | GbD | Delay discounting |
|  |  | AUD | Delay discounting |
| Amlung 2019 [12] | 1++ | BED | Delay discounting |
| Andrade 2024 [13] | 2+ | GmD | Impulsivity |
| Antons 2023 [14] | 2+ | GmD | Response inhibition (GNG, SST), Attentional inhibition impairments (Stroop) |
| Argyriou 2017 [15] | 1++ | GmD | Attentional inhibition impairments, Response inhibition |
| Balodis 2015 [16] | 2- | BED | Grey matter alterations, Impaired brain activity (fMRI) |
| Banks 2020 [17] | 2+ | GbD | Any substance use disorder, Antisocial personality disorder, Delinquency and/or criminal activities, Anxiety disorder, Depression, Conduct disorder, Alcohol, Cannabis |
| Benchebra 2019 [18] | 2+ | GmD | Poor physical health and chronic medical conditions |
| Blinn-Pike 2007 [19] | 2+ | GbD | Gender |
| Blume 2019 [20] | 2++ | BED | EEG abnormalities |
| Bogusz 2021 [21] | 1++ | BED | Alcohol |
| Bora 2017 [22] | 1++ | AUD | Beliefs/superstitions |
| Bretaña 2025 [23] | 2+ | BED | Domestic violence victimisation |
| Burleigh 2019 [24] | 2+ | GmD | Alcohol |
| Burleigh 2020 [25] | 2+ | GmD | EEG abnormalities |
| Calado 2016 [26] | 2+ | GbD | Gender, Age, Ethnicity, Marital status, Education |
| Calado 2017 [27] | 2+ | GbD | Gender, Ethnicity, Not living with both parents, Any substance use disorder |
| Calero-Elvira 2009 [28] | 1++ | BED | Illicit drug |
| Carr 2021 [29] | 2+ | BED | Response inhibition, Delay discounting, Cognitive flexibility, Attentional bias/task set-shifting, Impaired brain activity (fMRI) |
| Carter 1999 [30] | 1++ | AUD | Peripheral response to addiction-related cues |
| Casale 2021 [31] | 2++ | GmD | Metacognitive deficits |
| Caslini 2016 [32] | 1++ | BED | History of physical, sexual or emotional abuse |
| Castillo-Carniglia 2019 [33] | 1++ | AUD | Childhood ADHD |
| Chami 2019 [34] | 2++ | BED | EEG abnormalities |
| Chang 2023 [35] | 2+ | GmD | Anxiety, Depression, ADHD, Low self-esteem, Alexithymia, Negative affects, Neuroticism (more), Impulsivity, Delay discounting, Metacognitive deficits, Stressful life events, Ethnicity (minorities), Social support (low) |
| Cheng 2018 [36] | 1++ | GmD | Any mental health disorder |
| Cheng 2018 [37] | 1++ | GmD | Suicidal ideation |
| Chen 2020 [38] | 1++ | AUD | Probability discounting |
| Chew 2022 [39] | 1++ | GmD | Agreeableness, Conscientiousness, Extraversion, Neuroticism, Openness to experience |
| Choi 2021 [40] | 2+ | GmD | Grey matter alterations, Impaired brain activity (fMRI) |
| Chou 2011 [41] | 1+++ | AUD | Social support |
| Chowdhury 2017 [42] | 1+ | GbD | Response inhibition |
| Chung 2021 [43] | 1++ | GmD | Probability discounting |
| Clark 2016 [44] | 1+ | WA | Gender, Age, Education, Marital status, Agreeableness, Conscientiousness, Extraversion, Neuroticism, Openness to experience, Negative affects, Low self-esteem |
| Codling 2015 [45] | 2+ | CSBD | Gender, Peripheral response to addiction-related cues |
| Colledge 2020 [46] | 2++ | EA | Anxiety disorder, Depression, Alcohol, Tobacco/nicotine |
| Colton 2023 [47] | 1+ | BED | Decision-making impairments (IGT), Delay discounting, Response inhibition (GNG), Attentional inhibition impairment (Stroop), Cognitive flexibility |
| Conti 2017 [48] | 2++ | BED | Suicidal ideation |
| Conway 2016 [49] | 1+++ | AUD | Illicit drug |
| Coskunpinar 2014 [50] | 1++ | AUD | Low premeditation, Negative urgency |
| Coutelle 2024 [51] | 2+ | GmD | ADHD, Learning disorders, Anxiety, Depression, Suicidal ideation |
| Cudzik 2026 [52] | 2++ | BED | Alexithymia |
| Cury 2020 [53] | 1++ | BED | Attentional inhibition impairments, Response inhibition, Decision-making impairments, Cognitive flexibility |
| Darvishi 2015 [54] | 1++ | AUD | Suicidal ideation |
| Day 2015 [55] | 2+ | AUD | Learning disorders |
| de Alarcón 2019 [56] | 2+ | CSBD | Gender, Age, Anxiety disorder, Any substance use disorder, Alcohol, Tobacco/nicotine, Impulsivity, Cognitive flexibility, Emotion regulation deficit, Negative affects, Peripheral response to addiction-related cues, EEG abnormalities, Grey matter alterations, White matter alterations, Impaired brain activity (fMRI) |
| Dora 2023 [57] | 1++ | AUD | Negative affects |
| Dowling 2015 [58] | 1++ | GbD | Tobacco/nicotine, Cannabis, Bipolar disorder, Anxiety disorder, PTSD, Any mental health disorder, Depression, ADHD, Alcohol, Any substance use disorder |
| Dowling 2016 [59] | 2+ | GbD | Domestic violence victimisation |
| Dowling 2017 [60] | 1++ | GbD | Gender, Antisocial behaviours, Anxiety disorder, ADHD, Impulsivity, Peer antisocial behaviours, Perpetration of violence on partner or children, Alcohol, Cannabis, Education, Socio-economic status, Parent supervision, Suicidal ideation, Depression, Tobacco/nicotine, Illicit drug, Negative affects |
| Dullur 2021 [61] | 2++ | GmD | ADHD |
| Dumitru 2018 [62] | 2+ | EA | Gender |
| Durdle 2008 [63] | 1++ | GbD | Obsessive-compulsive traits |
| Erevik 2022 [64] | 2++ | GmD | Suicidal ideation, History of suicide attempt |
| Erskine 2018 [65] | 1+ | BED | Gender |
| Estupiñá 2024 [66] | 2++ | GmD | Emotion regulation deficit |
| Farrell 2003 [67] | 1+++ | AUD | Socio-economic status |
| Farstad 2016 [68] | 2- | BED | Neuroticism, Negative urgency |
| Félix 2025 [69] | 2+ | BED | Emotion regulation deficit, Emotion regulation strategy |
| Fernandes 2018 [70] | 1++, 2++ | BED | Alexithymia, Emotion regulation strategy |
| Flores 2025 [71] | 1++ | BED | Tobacco |
| Foran 2008 [72] | 1+ | AUD | Perpetration of violence on partner or children |
| Gadalla 2007 [73] | 1+ | BED | Alcohol |
| Gao 2021 [74] | 1++ | GmD | Grey matter alterations, Impaired brain activity (fMRI) |
| Gao 2022 [75] | 1+ | GmD | Depression, Anxiety, Emotional distress, Low self-esteem, ADHD, Alcohol drinking, Suicidal ideation |
| Gaudet 2025 [76] | 2++ | CSBD | Impulsivity (in general), Negative urgency, Low premeditation |
| Gervasi 2017 [77] | 2+ | GmD | Neuroticism, Impulsivity |
| Gianni 2020 [78] | 2+ | BED | Dopamine dysregulation in the brain |
| Giel 2017 [79] | 2+ | BED | Response inhibition, Attentional bias/task set-shifting |
| Gisbert-Pérez 2024 [80] | 1++ | GmD | Emotion regulation deficit |
| Godoy-Izquierdo 2021 [81] | 2+ | EA | Gender |
| Gola 2018 [82] | 2+ | CSBD | Peripheral response to addiction-related cues |
| González-Bueso 2018 [83] | 2++ | GmD | Anxiety disorder, Depression, ADHD |
| Gorrell 2021 [84] | 2++ | EA | Depression, Negative affects |
| Grant 2015 [85] | 1+++ | AUD | Gender, Age, Ethnicity, Education, Social functioning, Marital status, Any mental health disorder, Low level of perceived health, Anxiety disorder, PTSD, Bipolar disorder, Depression, Any substance use disorder, Tobacco/nicotine, Antisocial personality disorder |
| Grant Weinandy 2022 [86] | 2++ | CSBD | Anxiety disorder |
| Grogan 2020 [87] | 2++ | BED | Stressful life events |
| Guglielmucci 2019 [88] | 2+ | GmD | Dissociation |
| Guillaume 2015 [89] | 1++ | BED | Decision-making impairments |
| Guillou Landreat 2019 [90] | 2++ | GbD | Gender, Any substance use disorder, Socio-economic status, Any mental health disorder, Poor physical health and chronic medical conditions, Cognitive bias/distorted cognitions, Beliefs/superstitions |
| Gyollai 2014 [91] | 2+ | GbD | Contribution of genetic factors (twin studies), Associated alleles of dopamine gene(s) |
| Hamidovic 2019 [92] | 1++ | AUD | EEG abnormalities |
| Hamonniere 2018 [93] | 2+ | AUD | Metacognitive deficits |
|  |  | GbD | Metacognitive deficits |
| Hermand 2020 [94] | 2+ | CSBD | History of physical, sexual or emotional abuse, History of suicide attempt, Anxiety disorder, PTSD, Depression, Agreeableness, Conscientiousness, Extraversion, Neuroticism, Openness to experience, Dissociation |
| Honkalampi 2022 [95] | 1++ | AUD | Alexithymia |
| Hønsi 2013 [96] | 2+ | GbD | Attentional bias/task set-shifting |
| Hughes 2017 [97] | 1++ | AUD | History of physical, sexual or emotional abuse, Domestic violence victimisation |
| Huryk 2021 [98] | 2++ | BED | Education, Socio-economic status |
| Iceta 2021 [99] | 1++ | BED | Response inhibition, Decision-making impairments, Cognitive flexibility |
| Ioannidis 2019 [100] | 1+ | GbD | Response inhibition, Delay discounting, Attentional inhibition impairments, Decision-making impairments |
| Jennings 2021 [101] | 2+ | CSBD | Gender |
| Ji 2022 [102] | 1++ | GmD | Gender, Social support, Not living with both parents, Parent supervision, Stressful life events, Anxiety disorder, Depression, Agreeableness, Conscientiousness, Extraversion, Neuroticism, Disinhibition, Impulsivity |
| Juwono 2021 [103] | 2++ | EA | Gender, Social support, Anxiety disorder, Depression |
| Karaca 2017 [104] | 2+ | GbD | ADHD |
|  |  | BED | ADHD |
|  |  | CSBD | ADHD |
| Karila 2014 [105] | 2- | CSBD | Stressful life events, Any mental health disorder, Anxiety disorder, PTSD, ADHD, Any substance use disorder, Alcohol, Tobacco/nicotine, Illicit drug, Impulsivity, Negative affects, Low self-esteem |
| Kavanagh 2023 [106] | 1++ | GmD | Low self-esteem |
| Keegan 2021 [107] | 1++ | BED | Attentional bias/task set-shifting |
| Kenyhercz 2024 [108] | 1++ | WA | Social functioning |
| Kerr-Gaffney 2018 [109] | 2++ | BED | Attentional bias/task set-shifting |
| Kessler 1997 [110] | 1+++ | AUD | Antisocial behaviours |
| Keyes 2011 [111] | 2- | AUD | Stressful life events |
| Kim 2022 [112] | 1++ | GmD | Gender, Age |
| Kittel 2015 [113] | 2+ | BED | Response inhibition, Decision-making impairments, Delay discounting, Cognitive flexibility, Alexithymia, Impaired brain activity (fMRI) |
| Koncz 2023 [114] | 1++ | GmD | ADHD |
| Korchia 2022 [115] | 1++ | CSBD | ADHD |
| Kostro 2014 [116] | 2+ | BED | History of suicide attempt |
| Kovács 2017 [117] | 1++ | GbD | Decision-making impairments |
|  |  | AUD | Decision-making impairments |
| Kowalewska 2020 [118] | 2+ | CSBD | History of physical, sexual or emotional abuse, ADHD, Neuroticism, Impulsivity, Negative affects |
| Kowalewska 2025 [119] | 2- | CSBD | Gender, History of physical, sexual or emotional abuse, Age, Marital status, Impulsivity (in general), Emotion regulation deficit, Obsessive-compulsive traits, Depression, Anxiety |
| Kowatch 2005 [120] | 1+ | CSBD | Bipolar disorder |
| Krauss 2023 [121] | 1++ | BED | Low self-esteem |
| Kryszajtys 2018 [122] | 2+ | GbD | Delinquency and/or criminal activities |
| Kumar Sharma 2026 [123] | 2- | CBSD | Anxiety, Depression, Self-esteem (low) |
| Kun 2020 [124] | 1++ | WA | Anxiety disorder, Obsessive-compulsive traits, Agreeableness, Conscientiousness, Extraversion, Neuroticism, Openness to experience, Impulsivity, Negative affects, Low self-esteem |
| Kürbitz 2021 [125] | 2+ | CSBD | History of physical, sexual or emotional abuse, Any mental health disorder, Anxiety disorder, Depression, Any substance use disorder, Obsessive-compulsive traits, Neuroticism, Emotion regulation deficit, Emotional distress, Negative affects |
| Kuss 2018 [126] | 2- | GmD | EEG abnormalities, Grey matter alterations, Impaired brain activity (fMRI), Dopamine DRD2 dysfunction (PET) |
| Kyonka 2018 [127] | 1++ | GbD | Probability discounting |
| Lai 2022 [128] | 1+++ | AUD | Contribution of genetic factors (twin studies) |
| Lane 2016 [129] | 2+ | GbD | History of physical, sexual or emotional abuse, Perpetration of violence on partner or children |
| Latella 2021 [130] | 2+ | CSBD | Traumatic brain injury, Parkinson's disease |
| Leeman 2019 [131] | 2+ | CSBD | Impulsivity |
| Legault 2021 [132] | 2+ | GmD | Attentional inhibition impairments, Response inhibition, Attentional bias/task set-shifting, Impaired brain activity (fMRI) |
| Lemenager 2020 [133] | 2+ | GmD | Low self-esteem |
| Li 2021 [134] | 1++ | AUD | Grey matter alterations |
| Lo 2025 [135] | 1++ | GmD | History of physical, sexual or emotional abuse |
| Loo 2019 [136] | 2+ | GbD | Gender, Ethnicity, Age, Poor physical health and chronic medical conditions, History of physical, sexual or emotional abuse, Any mental health disorder, Anxiety disorder, Any substance use disorder |
| Lopez-Quintero 2011 [137] | 1+++ | AUD | ADHD, Conduct disorder, Cannabis, Cocaine, Marijuana |
| Lorains 2011 [138] | 1+ | GbD | Any substance use disorder, Illicit drug, Tobacco/nicotine, Anxiety disorder, Bipolar disorder, Depression, Alcohol, Antisocial personality disorder |
| Lui 2022 [139] | 1+, 1++ | AUD | Obsessive-compulsive traits, Agreeableness, Conscientiousness, Extraversion, Neuroticism, Openness to experience, Disinhibition |
| Luijten 2014 [140] | 2+ | AUD | Impaired brain activity (fMRI) |
| Luijten 2017 [141] | 1+ | GbD | Impaired brain activity (fMRI) |
| Lyssenko 2018 [142] | 1++ | AUD | Dissociation |
| MacLaren 2011 [143] | 1+ | GbD | Negative urgency, Disinhibition, Low premeditation, Negative affects |
| Manfredi 2021 [144] | 2++ | BED | Associated alleles of dopamine gene(s) |
| Männikkö 2020 [145] | 1++, 2+ | GmD | Anxiety disorder, Depression, ADHD, Neuroticism, Impulsivity, Low self-esteem |
| Maraz 2016 [146] | 1++ | CBSD | Gender, Age |
| Marchetti 2019 [147] | 2+ | GbD | Alexithymia |
| Marchica 2019 [148] | 2+ | GbD | Emotion regulation deficit |
|  |  | GmD | Emotion regulation deficit, Emotion regulation strategy |
| Marciano 2020 [149] | 1++ | GmD | Neuroticism |
| Mason 2021 [150] | 2++ | BED | Alexithymia, Emotion regulation deficit |
| McClelland 2016 [151] | 2+ | BED | Delay discounting |
| McDonald 2019 [152] | 2++ | BED | Bipolar disorder |
| McPhee 2023 [153] | 1++ | AUD | Response inhibition |
| Meng 2014 [154] | 1+ | GbD | Impaired brain activity (fMRI) |
| Meng 2015 [155] | 1+ | GmD | Impaired brain activity (fMRI) |
| Meque 2019 [156] | 1++ | AUD | Internalizing symptoms |
| Meyer 2011 [157] | 2- | EA | Obsessive-compulsive traits |
| Mihara 2017 [158] | 2+ | GmD | Gender, Age, Education, Marital status, Anxiety disorder, Depression, ADHD, Conduct disorder, Any substance use disorder, Agreeableness, Extraversion, Neuroticism, Impulsivity, Low self-esteem |
| Mitchell 2022 [159] | 1++ | AUD | Parkinson's disease |
| Moccia 2017 [160] | 2+ | GbD | Impaired brain activity (fMRI) |
| Molde 2018 [161] | 1++ | GbD | Parkinson's disease |
|  |  | BED | Parkinson's disease |
|  |  | CSBD | Parkinson's disease |
|  |  | CBSD | Parkinson's disease |
| Montgomery-Graham 2017 [162] | 2+ | CSBD | History of physical, sexual or emotional abuse, Anxiety disorder, Bipolar disorder, Depression, Any substance use disorder, Poor physical health and chronic medical conditions, Parkinson's disease |
| Monserrat Hernández 2024 [163] | 2++ | BED | Associated alleles of dopamine gene(s) |
| Moore 2019 [164] | 1+++ | AUD | Delinquency and/or criminal activities |
| Moore 2021 [165] | 2+ | GbD | Perpetration of violence on partner or children, Stressful life events, Domestic violence victimisation, History of physical, sexual or emotional abuse, PTSD |
| Mora-Salgueiro 2021 [166] | 2++ | GbD | Education, Marital status, Age, Gender, Any substance use disorder |
| Morkeviciute 2021 [167] | 1+ | WA | Agreeableness, Conscientiousness, Neuroticism, Openness to experience |
| Nakum 2016 [168] | 2+ | CSBD | Gender, Depression, Parkinson's disease |
| Nazar 2016 [169] | 1++ | BED | ADHD |
| Ngetich 2024 | 2+ | GmD | Learning disorders |
|  |  | CSBD | Learning disorders |
| Nicholls 2016 [170] | 2++ | BED | Depression |
| Nielsen 2019 [171] | 2+ | GmD | Parent supervision |
| Nielsen 2020 [172] | 2+ | GmD | History of physical, sexual or emotional abuse |
| Niu 2022 [173] | 1+ | GmD | Brain anatomy, Impaired brain activity |
| Nordmyr 2020 [174] | 2+ | GbD | Social support |
| Nowak 2018 [175] | 1++ | GbD | Gender, Age, Ethnicity |
| Nowakowski 2013 [176] | 2+ | BED | Alexithymia |
| Nuske 2026 [177] | 1++ | GmD | Impulsivity, Negative urgency, Lack of premeditation |
| Nuyens 2017 [178] | 2+ | GmD | Attentional inhibition impairments, Response inhibition, Delay discounting, Probability discounting |
| Olguin 2017 [179] | 2+ | BED | Poor physical health and chronic medical conditions |
| Palmisano 2016 [180] | 2+ | BED | Stressful life events, History of physical, sexual or emotional abuse, PTSD |
| Paulus 2018 [181] | 2- | GmD | Gender, Education, Socio-economic status, Marital status, Parent supervision, Stressful life events, Anxiety disorder, Depression, Impulsivity, Delay discounting, Negative affects, Low self-esteem, Impaired brain activity (fMRI), Dopamine dysregulation in the brain |
| Pelzer 2025 [182] | 2++ | BED | Attentional bias |
| Petrescu 2025 [183] | 2+ | GmD | Not living with both parents, Socio-economic status |
| Pettorruso 2020 [184] | 2++ | GbD | Associated alleles of dopamine gene(s), Dopamine dysregulation in the brain, Dopamine DRD2 dysfunction (PET), Dopamine transporter DAT dysfunction (SPECT) |
| Pinna 2015 [185] | 2- | CBSD | Gender, Age, Anxiety disorder, Any substance use disorder |
| Pirwani 2025 [186] | 2+ | EA | Brain anatomy, Impaired brain activity |
| Prefit 2019 [187] | 1++ | BED | Emotion regulation deficit, Emotion regulation strategy |
| Prunell-Castañé 2021 [188] | 1+ | BED | Attentional inhibition impairments, Response inhibition, Cognitive flexibility |
| Punia 2021 [189] | 2- | GbD | Cannabis |
| Qin 2020 [190] | 1++ | GbD | Grey matter alterations |
|  |  | GmD | Grey matter alterations |
| Quaglieri 2020 [191] | 1+ | GbD | Impaired brain activity (fMRI) |
| Raimo 2021 [192] | 1++, 2++ | GbD | Impaired brain activity (fMRI), Microstructural damage in bilateral superior longitudinal fascicle, corpus callosum and corticospinal tract, Grey matter alterations, White matter alterations |
| Ralph-Nearman 2019 [193] | 2++ | BED | Attentional bias/task set-shifting, Impaired brain activity (fMRI) |
| Raybould 2021 [194] | 2+ | GbD | Socio-economic status, Age, Ethnicity |
| Refahi 2025 [195] | 2++ | GmD | Attentional bias |
| Richard 2020 [196] | 2+ | GbD | Depression, Conduct disorder |
|  |  | GmD | Depression, Conduct disorder |
| Rizk 2020 [197] | 2+ | EA | Anxiety disorder, Depression, Low self-esteem |
| Roerecke 2014 [198] | 1++ | AUD | Poor physical health and chronic medical conditions |
| Rogier 2021 [199] | 1++ | GbD | Dissociation |
| Rogier 2021 [200] | 1++ | GbD | Metacognitive deficits |
| Rooke 2008 [201] | 1++ | AUD | Cognitive bias/distorted cognitions |
| Ropovik 2023 [202] | 1++ | GmD | Depression, Gender (being male), Anxiety, Impulsivity, Education, Low self-esteem, ADHD |
| Şalvarli 2021 [203] | 2++ | GmD | Agreeableness, Conscientiousness, Extraversion, Neuroticism, Openness to experience, Disinhibition |
| Şalvarli 2022 [204] | 2++ | GmD | Impulsivity |
| Schacht 2013 [205] | 1+ | AUD | Impaired brain activity (fMRI) |
| Schettler 2022 [206] | 2+ | GmD | Grey matter alterations, Impaired brain activity (fMRI) |
| Schneider 2017 [207] | 2+ | GmD | Socio-economic status, Not living with both parents, Parent supervision, History of physical, sexual or emotional abuse |
| Schultz 2014 [208] | 1++ | CSBD | Depression |
| Senra 2024 [209] | 1++ | BED | Obsessive-compulsive traits |
| Sharman 2019 [210] | 2+ | GbD | Education, Socio-economic status, Social support, Social functioning, Any mental health disorder, Low level of perceived health, Depression, Gender, Age, Ethnicity, Marital status, Stressful life events, History of physical, sexual or emotional abuse, Domestic violence victimisation, Anxiety disorder, Impulsivity, Emotional distress, Childhood ADHD, Any substance use disorder, Beliefs/superstitions, Conduct disorder, Antisocial behaviours, Traumatic brain injury, Negative affects, Low self-esteem, Learning disorders |
| Sheehan 2015 [211] | 2- | BED | Any mental health disorder, Anxiety disorder, Bipolar disorder, Depression, Any substance use disorder, Alcohol, Illicit drug, Poor physical health and chronic medical conditions, Obsessive-compulsive traits, Low self-esteem |
| Simon-Grima 2019 [212] | 1++ | EA | Age, Any mental health disorder, Poor physical health and chronic medical conditions |
| Slavin 2020 [213] | 2+ | CSBD | History of physical, sexual or emotional abuse |
| Slavin 2025 [214] | 2+ | CSBD | History of physical, sexual or emotional abuse |
| Smith 2018 [215] | 2++ | BED | Response inhibition, Decision-making impairments, Delay discounting, Attentional bias/task set-shifting |
| Sohn 2023 [216] | 1++ | BED | Attempted suicide |
| Sol-Gámez 2025 [217] | 2+ | CBSD | Gender, Age, Neuroticism, Self-esteem (low), Anxiety, Substance abuse |
| Soldati 2021 [218] | 2+ | CSBD | ADHD |
| Solmi 2016 [219] | 1++ | BED | Tobacco/nicotine |
| Spindler 2022 [220] | 1++ | AUD | Microstructural damage in bilateral superior longitudinal fascicle, corpus callosum and corticospinal tract |
| Spurrier 2014 [221] | 2+ | GbD | Cognitive bias/distorted cognitions |
| Starcke 2018 [222] | 1++ | GbD | Impaired brain activity (fMRI), Peripheral response to addiction-related cues, EEG abnormalities |
|  |  | GmD | EEG abnormalities, Impaired brain activity (fMRI) |
|  |  | CBSD | Peripheral response to addiction-related cues |
| Stellern 2023 [223] | 1++ | AUD | Emotion regulation deficit, Emotion regulation strategy |
| Stephan 2017 [224] | 1++ | AUD | Impulsivity, Attentional inhibition impairments, Cognitive flexibility, Attentional bias/task set-shifting, Probability discounting |
| Stevens 2021 [225] | 1++ | GmD | Gender, Age |
| Stojek 2017 [226] | 2+ | BED | Delay discounting |
| Stojek 2018 [227] | 2- | BED | Attentional bias/task set-shifting |
| Strømme 2021 [228] | 1++ | GbD | Openness to experience, Neuroticism, Conscientiousness, Agreeableness, Extraversion |
| Subramaniam 2015 [229] | 2+ | GbD | Education, Social support, Social functioning, Low level of perceived health, Anxiety disorder, Alcohol, Obsessive-compulsive traits, Age, Gender, Marital status, Ethnicity, Tobacco/nicotine, Poor physical health and chronic medical conditions |
| Sugaya 2019 [230] | 2+ | GmD | Depression, Conduct disorder, Antisocial behaviours, Impulsivity, Emotional distress, Associated alleles of dopamine gene(s) |
| Suresh 2023 [231] | 1+ | AUD | Associated alleles of dopamine gene(s) |
| Sussman 2011 [232] | 2+ | CSBD | Any substance use disorder, Alcohol, Tobacco/nicotine, Illicit drug |
|  |  | CBSD | Any substance use disorder |
| Szinay 2019 [233] | 1+++ | AUD | Low self-esteem |
| Tael-Öeren 2019 [234] | 1++ | AUD | Parent supervision |
| Theisejans 2025 [235] | 2- | CBSD | Impaired brain activity, Attentional bias, Response inhibition, Delay discounting, Decision-making impairments (IGT) |
| Theule 2019 [236] | 1+ | GbD | ADHD |
| Thiebaut 2019 [237] | 2+ | BED | Bipolar disorder |
| Thomas 2023 [238] | 2- | CBSD | Attentional bias, Cognitive flexibility, Decision-making impairments (IGT), Response inhibition |
| Trott 2020 [239] | 2++ | EA | Low self-esteem |
| Tse 2012 [240] | 2+ | GbD | Alcohol, Age, Marital status |
| Unsworth 2017 [241] | 1++ | AUD | Traumatic brain injury |
| van Timmeren 2018 [242] | 1++ | GbD | Attentional inhibition impairments, Cognitive flexibility, Attentional bias/task set-shifting |
| Van Tu 2020 [243] | 2+ | BED | History of physical, sexual or emotional abuse |
| Vandenberg 2022 [244] | 2++ | GbD | Socio-economic status |
| Varo 2019 [245] | 2+ | GbD | Bipolar disorder |
|  |  | CSBD | Any mental health disorder, Anxiety disorder, Bipolar disorder, Any substance use disorder |
|  |  | CBSD | Bipolar disorder |
| Velotti 2021 [246] | 1++ | GbD | Emotion regulation deficit, Emotion regulation strategy |
| Verplaetse 2021 [247] | 1+++ | AUD | Emotional distress |
| Waltmann 2021 [248] | 2+ | BED | Attentional inhibition impairments, Response inhibition, Decision-making impairments, Delay discounting, Cognitive flexibility, Attentional bias/task set-shifting, Impaired brain activity (fMRI) |
| Wang 2026 [249] | 1++ | EA | Anxiety disorder, Depression, Obsessive-compulsive traits, Emotion regulation deficit |
| Weinsztok 2021 [250] | 1++, 2++ | GbD | Delay discounting |
|  |  | GmD | Delay discounting |
|  |  | CBSD | Delay discounting |
| Wéry 2017 [251] | 2+ | CSBD | Gender, Marital status, Stressful life events, History of physical, sexual or emotional abuse, Anxiety disorder, PTSD, Depression |
| Westwood 2017 [252] | 1++ | BED | Alexithymia |
| Wilfley 2016 [253] | 2- | BED | Social functioning, Anxiety disorder, PTSD, Depression, Any substance use disorder, Poor physical health and chronic medical conditions |
| Wu 2013 [254] | 1++ | BED | Response inhibition |
| Wu 2014 [255] | 1++ | BED | Attentional bias/task set-shifting |
| Wu 2016 [256] | 1++ | BED | Decision-making impairments |
| Xuan 2017 [257] | 1+ | GbD | Contribution of genetic factors (twin studies) |
| Xue 2025 [258] | 1+ | GmD | Impaired brain activity (fMRI) |
| Yang 2016 [259] | 1++ | AUD | Grey matter alterations |
| Yao 2017 [260] | 1+ | GmD | Grey matter alterations, Impaired brain activity (fMRI) |
| Yao 2022 [261] | 1++ | GmD | Decision-making impairments |
| Yazdi-Feyzabadi 2019 [262] | 2+ | AUD | Peer antisocial behaviours |
| Young 2013 [263] | 2++ | EA | Obsessive-compulsive traits |
| Yu 2019 [264] | 2+ | GbD | Socio-economic status, Delinquency and/or criminal activities, Cognitive bias/distorted cognitions, Low self-esteem, Gender, Age |
| Yu 2024 [265] | 1++ | GmD | EEG abnormalities |
| Zeng 2023 [266] | 1++ | AUD | Impaired brain activity (fMRI) |
| Zeng 2023 [267] | 1++ | AUD | Impaired brain activity (fMRI) |
| Zheng 2019 [268] | 1++ | GmD | Impaired brain activity (fMRI) |
| Zheng 1 2025 [269] | 1++ | GmD | Depression |
| Zheng 2 2025 [270] | 1++ | GmD | White matter alterations, Microstructural damage in corpus callosum |
| Zhuang 2023 [271] | 1++ | GmD | Anxiety, ADHD, Depression, Impulsivity, Low education level, Conscientiousness, Agreeableness, Self-esteem, Neuroticism, Extraversion, Openness, History of abuse by family, Social support, Parental supervision |
| *AUD: alcohol use disorder, BED: binge eating disorder, CBSD: compulsive buying-shopping disorder, CSBD: compulsive sexual behaviour disorder, EA: exercise addiction, GbD: gambling disorder, GmD: gaming disorder, LoE: level of evidence, WA: work addiction* | | | |
|  | | | |

References:

[1]	Adolphe A, Khatib L, van Golde C, Gainsbury SM, Blaszczynski A. Crime and Gambling Disorders: A Systematic Review. Journal of Gambling Studies 2019;35:395–414. https://doi.org/10.1007/s10899-018-9794-7.

[2]	Ágh T, Kovács G, Pawaskar M, Supina D, Inotai A, Vokó Z. Epidemiology, health-related quality of life and economic burden of binge eating disorder: A systematic literature review. Eating and Weight Disorders 2015;20:1–12. https://doi.org/10.1007/s40519-014-0173-9.

[3]	Alcaraz-Ibáñez M, Paterna A, Sicilia Á, Griffiths MD. Examining the reliability of the scores of self-report instruments assessing problematic exercise: A systematic review and meta-analysis. J Behav Addict 2022. https://doi.org/10.1556/2006.2022.00014.

[4]	Alexandre MCM, Colonetti T, Bavaresco DV, Simon CS, Dondossola ER, Uggioni MLR, et al. Evaluation of the dopaminergic system with positron-emission tomography in alcohol abuse: A systematic review. Psychiatry Res 2019;281:112542. https://doi.org/10.1016/j.psychres.2019.112542.

[5]	Allami Y, Hodgins DC, Young M, Brunelle N, Currie S, Dufour M, et al. A meta-analysis of problem gambling risk factors in the general adult population. Addiction 2021;116:2968–77. https://doi.org/10.1111/add.15449.

[6]	Álvarez Ruiz EM, Gutiérrez-Rojas L. Comorbidity of bipolar disorder and eating disorders. Rev Psiquiatr Salud Ment 2015;8:232–41. https://doi.org/10.1016/j.rpsm.2014.12.001.

[7]	Amendola S, Bernath J, Presaghi F, Waller G, Hengartner MP. Bidirectional relationship between gaming disorder, internalizing psychopathology, psychological distress, and well-being: A systematic review with meta-analysis of longitudinal studies. J Affect Disord 2025;383:480–93. https://doi.org/10.1016/j.jad.2025.04.108.

[8]	Amiri S, Behnezhad S. Alcohol use and risk of suicide: a systematic review and Meta-analysis. J Addict Dis 2020;38:200–13. https://doi.org/10.1080/10550887.2020.1736757.

[9]	Amiri S, Fathi-Ashtiani M, Sedghijalal A, Fathi-Ashtiani A. Parental divorce and offspring smoking and alcohol use: a systematic review and meta-analysis of observational studies. J Addict Dis 2021;39:388–416. https://doi.org/10.1080/10550887.2021.1886576.

[10]	Amiri S, Sabzehparvar M. Childhood maltreatment and the risk of eating disorders: a meta-analysis of observational studies. Neuropsychiatr 2025;39:36–46. https://doi.org/10.1007/s40211-024-00505-3.

[11]	Amlung M, Vedelago L, Acker J, Balodis I, MacKillop J. Steep delay discounting and addictive behavior: a meta-analysis of continuous associations. Addiction 2017;112:51–62. https://doi.org/10.1111/add.13535.

[12]	Amlung M, Marsden E, Holshausen K, Morris V, Patel H, Vedelago L, et al. Delay discounting as a transdiagnostic process in psychiatric disorders: A meta-analysis. JAMA Psychiatry 2019;76:1176–86. https://doi.org/10.1001/jamapsychiatry.2019.2102.

[13]	Andrade ALM, Lobato FBH, Stange N, Scatena A, de Oliveira WA, Kim HS, et al. The association between gaming disorder and impulsivity: A systematic review. Estudos de Psicologia 2024;41. https://doi.org/10.1590/1982-0275202441e220032.

[14]	Antons S, Mueller SM, Neumann P, Müller A, Steins-Loeber S. Stimuli-Specific Inhibitory Control in Disorders Due to Addictive Behaviours: a Review of Current Evidence and Discussion of Methodological Challenges. Current Addiction Reports 2023;10:749–69. https://doi.org/10.1007/s40429-023-00517-z.

[15]	Argyriou E, Davison CB, Lee TTC. Response inhibition and Internet Gaming Disorder: A meta-analysis. Addictive Behaviors 2017;71:54–60. https://doi.org/10.1016/j.addbeh.2017.02.026.

[16]	Balodis IM, Grilo CM, Potenza MN. Neurobiological features of binge eating disorder. CNS Spectr 2015;20:557–65. https://doi.org/10.1017/S1092852915000814.

[17]	Banks J, Waters J, Andersson C, Olive V. Prevalence of Gambling Disorder Among Prisoners: A Systematic Review. International Journal of Offender Therapy and Comparative Criminology 2020;64:1199–216. https://doi.org/10.1177/0306624X19862430.

[18]	Benchebra L, Alexandre J-M, Dubernet J, Fatséas M, Auriacombe M. Gambling and Gaming disorders and physical health of players: A critical review of the literature. Presse Medicale 2019;48:1551–68. https://doi.org/10.1016/j.lpm.2019.10.014.

[19]	Blinn-Pike L, Worthy SL, Jonkman JN. Disordered gambling among college students: A meta-analytic synthesis. Journal of Gambling Studies 2007;23:175–83. https://doi.org/10.1007/s10899-006-9036-2.

[20]	Blume M, Schmidt R, Hilbert A. Abnormalities in the EEG power spectrum in bulimia nervosa, binge‐eating disorder, and obesity: A systematic review. European Eating Disorders Review 2019;27:124–36. https://doi.org/10.1002/erv.2654.

[21]	Bogusz K, Kopera M, Jakubczyk A, Trucco EM, Kucharska K, Walenda A, et al. Prevalence of alcohol use disorder among individuals who binge eat: A systematic review and meta‐analysis. Addiction 2021;116:18–31. https://doi.org/10.1111/add.15155.

[22]	Bora E, Zorlu N. Social cognition in alcohol use disorder: a meta-analysis. Addiction 2017;112:40–8. https://doi.org/10.1111/add.13486.

[23]	Bretaña I, Mendia J, Díaz-Gorriti V, Rotaetxe O. Intimate partner violence and eating disorders: A systematic review. Current Psychology: A Journal for Diverse Perspectives on Diverse Psychological Issues 2025;44:1696–716. https://doi.org/10.1007/s12144-024-07164-1.

[24]	Burleigh T, Griffiths M, Sumich A, Stavropoulos V, Kuss D. A Systematic Review of the Co-occurrence of Gaming Disorder and Other Potentially Addictive Behaviors. CURRENT ADDICTION REPORTS 2019;6:383–401. https://doi.org/10.1007/s40429-019-00279-7.

[25]	Burleigh TL, Griffiths MD, Sumich A, Wang GY, Kuss DJ. Gaming disorder and Internet addiction: A systematic review of resting-state EEG studies. Addictive Behaviors 2020;107. https://doi.org/10.1016/j.addbeh.2020.106429.

[26]	Calado F, Griffiths MD. Problem gambling worldwide: An update and systematic review of empirical research (2000-2015). Journal of Behavioral Addictions 2016;5:592–613. https://doi.org/10.1556/2006.5.2016.073.

[27]	Calado F, Alexandre J, Griffiths MD. Prevalence of Adolescent Problem Gambling: A Systematic Review of Recent Research. Journal of Gambling Studies 2017;33:397–424. https://doi.org/10.1007/s10899-016-9627-5.

[28]	Calero-Elvira A, Krug I, Davis K, López C, Fernández-Aranda F, Treasure J. Meta-analysis on drugs in people with eating disorders. European Eating Disorders Review 2009;17:243–59. https://doi.org/10.1002/(ISSN)1099-096810.1002/erv.v17:410.1002/erv.936.

[29]	Carr MM, Wiedemann AA, Macdonald-Gagnon G, Potenza MN. Impulsivity and compulsivity in binge eating disorder: A systematic review of behavioral studies. Progress in Neuro-Psychopharmacology & Biological Psychiatry 2021;110. https://doi.org/10.1016/j.pnpbp.2021.110318.

[30]	Carter BL, Tiffany ST. Meta-analysis of cue-reactivity in addiction research. Addiction 1999;94:327–40.

[31]	Casale S, Musicò A, Spada MM. A systematic review of metacognitions in Internet Gaming Disorder and problematic Internet, smartphone and social networking sites use. Clin Psychol Psychother 2021;28:1494–508. https://doi.org/10.1002/cpp.2588.

[32]	Caslini M, Bartoli F, Crocamo C, Dakanalis A, Clerici M, Carrà G. Disentangling the association between child abuse and eating disorders: A systematic review and meta-analysis. Psychosomatic Medicine 2016;78:79–90. https://doi.org/10.1097/PSY.0000000000000233.

[33]	Castillo-Carniglia A, Keyes KM, Hasin DS, Cerdá M. Psychiatric comorbidities in alcohol use disorder. Lancet Psychiatry 2019;6:1068–80. https://doi.org/10.1016/S2215-0366(19)30222-6.

[34]	Chami R, Cardi V, Lautarescu A, Mallorquí-Bagué N, McLoughlin G. Neural responses to food stimuli among individuals with eating and weight disorders: A systematic review of event-related potentials. International Review of Psychiatry 2019;31:318–31. https://doi.org/10.1080/09540261.2019.1622515.

[35]	Chang RS, Lee M, Im JJ, Choi K-H, Kim J, Chey J, et al. Biopsychosocial factors of gaming disorder: a systematic review employing screening tools with well-defined psychometric properties. Front Psychiatry 2023;14:1200230. https://doi.org/10.3389/fpsyt.2023.1200230.

[36]	Cheng C, Cheung MW-L, Wang H. Multinational comparison of internet gaming disorder and psychosocial problems versus well-being: Meta-analysis of 20 countries. Computers in Human Behavior 2018;88:153–67. https://doi.org/10.1016/j.chb.2018.06.033.

[37]	Cheng Y-S, Tseng P-T, Lin P-Y, Chen T-Y, Stubbs B, Carvalho AF, et al. Internet Addiction and Its Relationship With Suicidal Behaviors: A Meta-Analysis of Multinational Observational Studies. J Clin Psychiatry 2018;79:17r11761. https://doi.org/10.4088/JCP.17r11761.

[38]	Chen S, Yang P, Chen T, Su H, Jiang H, Zhao M. Risky decision-making in individuals with substance use disorder: A meta-analysis and meta-regression review. Psychopharmacology (Berl) 2020;237:1893–908. https://doi.org/10.1007/s00213-020-05506-y.

[39]	Chew PKH. A meta-analytic review of Internet gaming disorder and the Big Five personality factors. Addict Behav 2022;126:107193. https://doi.org/10.1016/j.addbeh.2021.107193.

[40]	Choi E, Shin S-H, Ryu J-K, Jung K-I, Hyun Y, Kim J, et al. Association of extensive video gaming and cognitive function changes in brain-imaging studies of pro gamers and individuals with gaming disorder: Systematic literature review. JMIR Serious Games 2021;9. https://doi.org/10.2196/25793.

[41]	Chou K-L, Liang K, Sareen J. The association between social isolation and DSM-IV mood, anxiety, and substance use disorders: wave 2 of the National Epidemiologic Survey on Alcohol and Related Conditions. J Clin Psychiatry 2011;72:1468–76. https://doi.org/10.4088/JCP.10m06019gry.

[42]	Chowdhury NS, Livesey EJ, Blaszczynski A, Harris JA. Pathological Gambling and Motor Impulsivity: A Systematic Review with Meta-Analysis. Journal of Gambling Studies 2017;33:1213–39. https://doi.org/10.1007/s10899-017-9683-5.

[43]	Chung W, Sun C, Tsai I, Hung K, Chiu H, Tzang R, et al. A systematic review and meta-analysis on the clinical implications of probability discounting among individuals with Internet gaming disorder. SCIENTIFIC REPORTS 2021;11. https://doi.org/10.1038/s41598-021-82822-z.

[44]	Clark MA, Michel JS, Zhdanova L, Pui SY, Baltes BB. All work and no play? A meta-analytic examination of the correlates and outcomes of workaholism. Journal of Management 2016;42:1836–73. https://doi.org/10.1177/0149206314522301.

[45]	Codling D, Shaw P, David AS. Hypersexuality in Parkinson’s Disease: Systematic Review and Report of 7 New Cases. Movement Disorders Clinical Practice 2015;2:116–26. https://doi.org/10.1002/mdc3.12155.

[46]	Colledge F, Sattler I, Schilling H, Gerber M, Pühse U, Walter M. Mental disorders in individuals at risk for exercise addiction – A systematic review. Addict Behav Rep 2020;12. https://doi.org/10.1016/j.abrep.2020.100314.

[47]	Colton E, Wilson K-E, Chong TT-J, Verdejo-Garcia A. Dysfunctional decision-making in binge-eating disorder: A meta-analysis and systematic review. Neurosci Biobehav Rev 2023;152:105250. https://doi.org/10.1016/j.neubiorev.2023.105250.

[48]	Conti C, Lanzara R, Scipioni M, Iasenza M, Guagnano MT, Fulcheri M. The relationship between binge eating disorder and suicidality: A systematic review. Frontiers in Psychology 2017;8. https://doi.org/10.3389/fpsyg.2017.02125.

[49]	Conway KP, Swendsen J, Husky MM, He J-P, Merikangas KR. Association of Lifetime Mental Disorders and Subsequent Alcohol and Illicit Drug Use: Results From the National Comorbidity Survey-Adolescent Supplement. J Am Acad Child Adolesc Psychiatry 2016;55:280–8. https://doi.org/10.1016/j.jaac.2016.01.006.

[50]	Coskunpinar A, Dir AL, Cyders MA. Multidimensionality in impulsivity and alcohol use: a meta-analysis using the UPPS model of impulsivity. Alcohol Clin Exp Res 2013;37:1441–50. https://doi.org/10.1111/acer.12131.

[51]	Coutelle R, Balzer J, Rolling J, Lalanne L. Problematic gaming, psychiatric comorbidities, and adolescence: A systematic review of the literature. Addict Behav 2024;157:108091. https://doi.org/10.1016/j.addbeh.2024.108091.

[52]	Cudzik M, Lis M, Wichniak A, Bieńkowski P. Alexithymia among patients with obesity - systematic review. J Psychiatr Res 2026;193:519–27. https://doi.org/10.1016/j.jpsychires.2025.12.030.

[53]	Cury MEG, Berberian A, Scarpato BS, Kerr-Gaffney J, Santos FH, Claudino AM. Scrutinizing Domains of Executive Function in Binge Eating Disorder: A Systematic Review and Meta-Analysis. Front Psychiatry 2020;11:288. https://doi.org/10.3389/fpsyt.2020.00288.

[54]	Darvishi N, Farhadi M, Haghtalab T, Poorolajal J. Alcohol-related risk of suicidal ideation, suicide attempt, and completed suicide: a meta-analysis. PLoS One 2015;10:e0126870. https://doi.org/10.1371/journal.pone.0126870.

[55]	Day AM, Kahler CW, Ahern DC, Clark US. Executive Functioning in Alcohol Use Studies: A Brief Review of Findings and Challenges in Assessment. Curr Drug Abuse Rev 2015;8:26–40. https://doi.org/10.2174/1874473708666150416110515.

[56]	de Alarcón R, de la Iglesia JI, Casado NM, Montejo AL. Online porn addiction: What we know and what we don’t—a systematic review. Journal of Clinical Medicine 2019;8. https://doi.org/10.3390/jcm8010091.

[57]	Dora J, Piccirillo M, Foster KT, Arbeau K, Armeli S, Auriacombe M, et al. The daily association between affect and alcohol use: A meta-analysis of individual participant data. Psychol Bull 2023;149:1–24. https://doi.org/10.1037/bul0000387.

[58]	Dowling NA, Cowlishaw S, Jackson AC, Merkouris SS, Francis KL, Christensen DR. Prevalence of psychiatric co-morbidity in treatment-seeking problem gamblers: A systematic review and meta-analysis. Australian and New Zealand Journal of Psychiatry 2015;49:519–39. https://doi.org/10.1177/0004867415575774.

[59]	Dowling N, Suomi A, Jackson A, Lavis T, Patford J, Cockman S, et al. Problem Gambling and Intimate Partner Violence: A Systematic Review and Meta-Analysis. Trauma, Violence, and Abuse 2016;17:43–61. https://doi.org/10.1177/1524838014561269.

[60]	Dowling NA, Merkouris SS, Greenwood CJ, Oldenhof E, Toumbourou JW, Youssef GJ. Early risk and protective factors for problem gambling: A systematic review and meta-analysis of longitudinal studies. Clinical Psychology Review 2017;51:109–24. https://doi.org/10.1016/j.cpr.2016.10.008.

[61]	Dullur P, Krishnan V, Diaz AM. A systematic review on the intersection of attention-deficit hyperactivity disorder and gaming disorder. J Psychiatr Res 2021;133:212–22. https://doi.org/10.1016/j.jpsychires.2020.12.026.

[62]	Dumitru DC, Dumitru T, Maher AJ. A systematic review of exercise addiction: Examining gender differences. J Phys Educ Sport 2018;18:1738–47. https://doi.org/10.7752/jpes.2018.03253.

[63]	Durdle H, Gorey KM, Stewart SH. A meta-analysis examining the relations among pathological gambling, obsessive-compulsive disorder, and obsessive-compulsive traits. Psychological Reports 2008;103:485–98. https://doi.org/10.2466/PR0.103.2.485-498.

[64]	Erevik EK, Landrø H, Mattson ÅL, Kristensen JH, Kaur P, Pallesen S. Problem gaming and suicidality: A systematic literature review. Addict Behav Rep 2022;15:100419. https://doi.org/10.1016/j.abrep.2022.100419.

[65]	Erskine HE, Whiteford HA. Epidemiology of binge eating disorder. Curr Opin Psychiatry 2018;31:462–70. https://doi.org/10.1097/YCO.0000000000000449.

[66]	Estupiñá FJ, Bernaldo-de-Quirós M, Vallejo-Achón M, Fernández-Arias I, Labrador F. Emotional regulation in Gaming Disorder: A systematic review. Am J Addict 2024;33:605–20. https://doi.org/10.1111/ajad.13621.

[67]	Farrell M, Howes S, Taylor C, Lewis G, Jenkins R, Bebbington P, et al. Substance misuse and psychiatric comorbidity: an overview of the OPCS National Psychiatric Morbidity Survey. Int Rev Psychiatry 2003;15:43–9. https://doi.org/10.1080/0954026021000045930.

[68]	Farstad SM, McGeown LM, von Ranson KM. Eating disorders and personality, 2004–2016: A systematic review and meta-analysis. Clinical Psychology Review 2016;46:91–105. https://doi.org/10.1016/j.cpr.2016.04.005.

[69]	Félix S, Gonçalves S, Ramos R, Tavares A, Vaz AR, Machado PPP, et al. Emotion regulation as a transdiagnostic construct across the spectrum of disordered eating in adolescents: A systematic review. J Affect Disord 2025;369:868–85. https://doi.org/10.1016/j.jad.2024.10.017.

[70]	Fernandes J, Ferreira-Santos F, Miller K, Torres S. Emotional processing in obesity: a systematic review and exploratory meta-analysis. Obes Rev 2018;19:111–20. https://doi.org/10.1111/obr.12607.

[71]	Flores BEV, Pedro MOP, Perrotte G, Accari Pedrosa MA, Florence L, Florio L, et al. Current tobacco smoking prevalence in individuals living with eating disorders: A meta-analysis and meta-regression study. J Psychosom Res 2025;190:112047. https://doi.org/10.1016/j.jpsychores.2025.112047.

[72]	Foran HM, O’Leary KD. Alcohol and intimate partner violence: a meta-analytic review. Clin Psychol Rev 2008;28:1222–34. https://doi.org/10.1016/j.cpr.2008.05.001.

[73]	Gadalla T, Piran N. Co-occurrence of eating disorders and alcohol use disorders in women: A meta analysis. Archives of Women’s Mental Health 2007;10:133–40. https://doi.org/10.1007/s00737-007-0184-x.

[74]	Gao X, Zhang M, Yang Z, Wen M, Huang H, Zheng R, et al. Structural and Functional Brain Abnormalities in Internet Gaming Disorder and Attention-Deficit/Hyperactivity Disorder: A Comparative Meta-Analysis. Front Psychiatry 2021;12:679437. https://doi.org/10.3389/fpsyt.2021.679437.

[75]	Gao Y-X, Wang J-Y, Dong G-H. The prevalence and possible risk factors of internet gaming disorder among adolescents and young adults: Systematic reviews and meta-analyses. J Psychiatr Res 2022;154:35–43. https://doi.org/10.1016/j.jpsychires.2022.06.049.

[76]	Gaudet É, Teumeni FN, Popova N, Coulombe S, Demetrovics Z, Bőthe B. Compulsive Sexual Behavior and Problematic Pornography Use on the Impulsivity-Compulsivity Axis: A Systematic Review. Current Addiction Reports 2025;12. https://doi.org/10.1007/s40429-025-00637-8.

[77]	Gervasi AM, La Marca L, Costanzo A, Pace U, Guglielmucci F, Schimmenti A. Personality and Internet Gaming Disorder: a Systematic Review of Recent Literature. Curr Addict Rep 2017;4:293–307. https://doi.org/10.1007/s40429-017-0159-6.

[78]	Gianni AD, De Donatis D, Valente S, De Ronchi D, Atti AR. Eating disorders: Do PET and SPECT have a role? A systematic review of the literature. Psychiatry Research: Neuroimaging 2020;300. https://doi.org/10.1016/j.pscychresns.2020.111065.

[79]	Giel KE, Teufel M, Junne F, Zipfel S, Schag K. Food-Related Impulsivity in Obesity and Binge Eating Disorder-A Systematic Update of the Evidence. Nutrients 2017;9. https://doi.org/10.3390/nu9111170.

[80]	Gisbert-Pérez J, Badenes-Ribera L, Martí-Vilar M. Emotional intelligence and gaming disorder symptomatology: A systematic review and meta-analysis. Adolescent Research Review 2024;9:621–46. https://doi.org/10.1007/s40894-024-00233-3.

[81]	Godoy-Izquierdo D, Ramírez MJ, Díaz I, López-Mora C. A Systematic Review on Exercise Addiction and the Disordered Eating-Eating Disorders Continuum in the Competitive Sport Context. Int J Ment Health Addict 2021. https://doi.org/10.1007/s11469-021-00610-2.

[82]	Gola M, Draps M. Ventral Striatal Reactivity in Compulsive Sexual Behaviors. Frontiers in Psychiatry 2018;9. https://doi.org/10.3389/fpsyt.2018.00546.

[83]	González-Bueso V, Santamaría JJ, Fernández D, Merino L, Montero E, Ribas J. Association between internet gaming disorder or pathological video-game use and comorbid psychopathology: A comprehensive review. Int J Environ Res Public Health 2018;15. https://doi.org/10.3390/ijerph15040668.

[84]	Gorrell S, Flatt R, Bulik C, Le Grange D. Psychosocial etiology of maladaptive exercise and its role in eating disorders: A systematic review. INTERNATIONAL JOURNAL OF EATING DISORDERS 2021;54:1358–76. https://doi.org/10.1002/eat.23524.

[85]	Grant BF, Goldstein RB, Saha TD, Chou SP, Jung J, Zhang H, et al. Epidemiology of DSM-5 Alcohol Use Disorder: Results From the National Epidemiologic Survey on Alcohol and Related Conditions III. JAMA Psychiatry 2015;72:757–66. https://doi.org/10.1001/jamapsychiatry.2015.0584.

[86]	Grant Weinandy JT, Lee B, Hoagland KC, Grubbs JB, Bőthe B. Anxiety and Compulsive Sexual Behavior Disorder: A Systematic Review. J Sex Res 2022:1–13. https://doi.org/10.1080/00224499.2022.2066616.

[87]	Grogan K, MacGarry D, Bramham J, Scriven M, Maher C, Fitzgerald A. Family-related non-abuse adverse life experiences occurring for adults diagnosed with eating disorders: a systematic review. J Eat Disord 2020;8:36. https://doi.org/10.1186/s40337-020-00311-6.

[88]	Guglielmucci F, Monti M, Franzoi IG, Santoro G, Granieri A, Billieux J, et al. Dissociation in Problematic Gaming: a Systematic Review. Curr Addict Rep 2019;6. https://doi.org/10.1007/s40429-019-0237-z.

[89]	Guillaume S, Gorwood P, Jollant F, Van Den Eynde F, Courtet P, Richard-Devantoy S. Impaired decision-making in symptomatic anorexia and bulimia nervosa patients: A meta-analysis. Psychological Medicine 2015;45:3377–91. https://doi.org/10.1017/S003329171500152X.

[90]	Guillou Landreat M, Cholet J, Grall Bronnec M, Lalande S, Le Reste JY. Determinants of Gambling Disorders in Elderly People—A Systematic Review. Frontiers in Psychiatry 2019;10. https://doi.org/10.3389/fpsyt.2019.00837.

[91]	Gyollai A, Griffiths MD, Barta C, Vereczkei A, Urbán R, Kun B, et al. The genetics of problem and pathological gambling: A systematic review. Current Pharmaceutical Design 2014;20:3993–9. https://doi.org/10.2174/13816128113199990626.

[92]	Hamidovic A, Wang Y. The P300 in alcohol use disorder: A meta-analysis and meta-regression. Prog Neuropsychopharmacol Biol Psychiatry 2019;95:109716. https://doi.org/10.1016/j.pnpbp.2019.109716.

[93]	Hamonniere T, Varescon I. Metacognitive beliefs in addictive behaviours: A systematic review. Addictive Behaviors 2018;85:51–63. https://doi.org/10.1016/j.addbeh.2018.05.018.

[94]	Hermand M, Benyamina A, Donnadieu-Rigole H, Petillion A, Amirouche A, Romeo B, et al. Addictive Use of Online Sexual Activities and its Comorbidities: a Systematic Review. CURRENT ADDICTION REPORTS 2020;7:194–209. https://doi.org/10.1007/s40429-020-00301-3.

[95]	Honkalampi K, Jokela M, Lehto SM, Kivimäki M, Virtanen M. Association between alexithymia and substance use: A systematic review and meta-analysis. Scand J Psychol 2022;63:427–38. https://doi.org/10.1111/sjop.12821.

[96]	Hønsi A, Mentzoni RA, Molde H, Pallesen S. Attentional Bias in Problem Gambling: A Systematic Review. Journal of Gambling Studies 2013;29:359–75. https://doi.org/10.1007/s10899-012-9315-z.

[97]	Hughes K, Bellis MA, Hardcastle KA, Sethi D, Butchart A, Mikton C, et al. The effect of multiple adverse childhood experiences on health: a systematic review and meta-analysis. Lancet Public Health 2017;2:e356–66. https://doi.org/10.1016/S2468-2667(17)30118-4.

[98]	Huryk KM, Drury CR, Loeb KL. Diseases of affluence? A systematic review of the literature on socioeconomic diversity in eating disorders. Eat Behav 2021;43:101548. https://doi.org/10.1016/j.eatbeh.2021.101548.

[99]	Iceta S, Rodrigue C, Legendre M, Daoust J, Flaudias V, Michaud A, et al. Cognitive function in binge eating disorder and food addiction: A systematic review and three-level meta-analysis. Progress in Neuro-Psychopharmacology & Biological Psychiatry 2021;111. https://doi.org/10.1016/j.pnpbp.2021.110400.

[100]	Ioannidis K, Hook R, Wickham K, Grant JE, Chamberlain SR. Impulsivity in Gambling Disorder and problem gambling: a meta-analysis. Neuropsychopharmacology 2019;44:1354–61. https://doi.org/10.1038/s41386-019-0393-9.

[101]	Jennings TL, Lyng T, Gleason N, Finotelli I, Coleman E. Compulsive sexual behavior, religiosity, and spirituality: A systematic review. J Behav Addict 2021;10:854–78. https://doi.org/10.1556/2006.2021.00084.

[102]	Ji Y, Yin MXC, Zhang AY, Wong DFK. Risk and protective factors of Internet gaming disorder among Chinese people: A meta-analysis. Aust N Z J Psychiatry 2022;56:332–46. https://doi.org/10.1177/00048674211025703.

[103]	Juwono ID, Tolnai N, Szabo A. Exercise Addiction in Athletes: a Systematic Review of the Literature. Int J Ment Health Addict 2021. https://doi.org/10.1007/s11469-021-00568-1.

[104]	Karaca S, Saleh A, Canan F, Potenza MN. Comorbidity between Behavioral Addictions and Attention Deficit/Hyperactivity Disorder: a Systematic Review. International Journal of Mental Health and Addiction 2017;15:701–24. https://doi.org/10.1007/s11469-016-9660-8.

[105]	Karila L, Wéry A, Weinstein A, Cottencin O, Petit A, Reynaud M, et al. Sexual addiction or hypersexual disorder: different terms for the same problem? A review of the literature. Curr Pharm Des 2014;20:4012–20. https://doi.org/10.2174/13816128113199990619.

[106]	Kavanagh M, Brett C, Brignell C. What is the reported relationship between self-esteem and gaming disorder? A systematic review and meta-analysis. Computers in Human Behavior 2023;145. https://doi.org/10.1016/j.chb.2023.107776.

[107]	Keegan E, Tchanturia K, Wade TD. Central coherence and set‐shifting between nonunderweight eating disorders and anorexia nervosa: A systematic review and meta‐analysis. International Journal of Eating Disorders 2021;54:229–43. https://doi.org/10.1002/eat.23430.

[108]	Kenyhercz V, Mervó B, Lehel N, Demetrovics Z, Kun B. Work addiction and social functioning: A systematic review and five meta-analyses. PLoS One 2024;19:e0303563. https://doi.org/10.1371/journal.pone.0303563.

[109]	Kerr-Gaffney J, Harrison A, Tchanturia K. Eye-tracking research in eating disorders: A systematic review. Int J Eat Disord 2018. https://doi.org/10.1002/eat.22998.

[110]	Kessler RC, Crum RM, Warner LA, Nelson CB, Schulenberg J, Anthony JC. Lifetime co-occurrence of DSM-III-R alcohol abuse and dependence with other psychiatric disorders in the National Comorbidity Survey. Arch Gen Psychiatry 1997;54:313–21. https://doi.org/10.1001/archpsyc.1997.01830160031005.

[111]	Keyes KM, Hatzenbuehler ML, Hasin DS. Stressful life experiences, alcohol consumption, and alcohol use disorders: the epidemiologic evidence for four main types of stressors. Psychopharmacology (Berl) 2011;218:1–17. https://doi.org/10.1007/s00213-011-2236-1.

[112]	Kim HS, Son G, Roh E-B, Ahn W-Y, Kim J, Shin S-H, et al. Prevalence of gaming disorder: A meta-analysis. Addictive Behaviors 2022;126. https://doi.org/10.1016/j.addbeh.2021.107183.

[113]	Kittel R, Brauhardt A, Hilbert A. Cognitive and emotional functioning in binge‐eating disorder: A systematic review. International Journal of Eating Disorders 2015;48:535–54. https://doi.org/10.1002/eat.22419.

[114]	Koncz P, Demetrovics Z, Takacs ZK, Griffiths MD, Nagy T, Király O. The emerging evidence on the association between symptoms of ADHD and gaming disorder: A systematic review and meta-analysis. Clin Psychol Rev 2023;106:102343. https://doi.org/10.1016/j.cpr.2023.102343.

[115]	Korchia T, Boyer L, Deneuville M, Etchecopar-Etchart D, Lancon C, Fond G. ADHD prevalence in patients with hypersexuality and paraphilic disorders: a systematic review and meta-analysis. Eur Arch Psychiatry Clin Neurosci 2022. https://doi.org/10.1007/s00406-022-01421-9.

[116]	Kostro K, Lerman JB, Attia E. The current status of suicide and self-injury in eating disorders: A narrative review. J Eating Disord 2014;2. https://doi.org/10.1186/s40337-014-0019-x.

[117]	Kovács I, Richman MJ, Janka Z, Maraz A, Andó B. Decision making measured by the Iowa Gambling Task in alcohol use disorder and gambling disorder: a systematic review and meta-analysis. Drug and Alcohol Dependence 2017;181:152–61. https://doi.org/10.1016/j.drugalcdep.2017.09.023.

[118]	Kowalewska E, Gola M, Kraus SW, Lew-Starowicz M. Spotlight on compulsive sexual behavior disorder: A systematic review of research on women. Neuropsychiatric Disease and Treatment 2020;16. https://doi.org/10.2147/NDT.S221540.

[119]	Kowalewska E, Szumska I, Lew-Starowicz M. Expanding the Lens: A Systematic Review of the Latest Research on Compulsive Sexual Behavior and Problematic Pornography Use among Women. Current Addiction Reports 2025;12. https://doi.org/10.1007/s40429-025-00674-3.

[120]	Kowatch RA, Youngstrom EA, Danielyan A, Findling RL. Review and meta-analysis of the phenomenology and clinical characteristics of mania in children and adolescents. Bipolar Disorders 2005;7:483–96. https://doi.org/10.1111/j.1399-5618.2005.00261.x.

[121]	Krauss S, Dapp LC, Orth U. The link between low self-esteem and eating disorders: A meta-analysis of longitudinal studies. Clinical Psychological Science 2023;11:1141–58. https://doi.org/10.1177/21677026221144255.

[122]	Kryszajtys DT, Hahmann TE, Schuler A, Hamilton-Wright S, Ziegler CP, Matheson FI. Problem gambling and delinquent behaviours among adolescents: A scoping review. Journal of Gambling Studies 2018;34:893–914. https://doi.org/10.1007/s10899-018-9754-2.

[123]	Kumar Sharma BK, Kundu S, Rana S, Pathak A. Compulsive Buying Behavior: A Review and Research Agenda. International Journal of Consumer Studies 2026;50. https://doi.org/10.1111/ijcs.70163.

[124]	Kun B, Takacs ZK, Richman MJ, Griffiths MD, Demetrovics Z. Work addiction and personality: A meta-analytic study. J Behav Addict 2020;9:945–66. https://doi.org/10.1556/2006.2020.00097.

[125]	Kürbitz LI, Briken P. Is compulsive sexual behavior different in women compared to men? J Clin Med 2021;10. https://doi.org/10.3390/jcm10153205.

[126]	Kuss DJ. Policy, prevention, and regulation for Internet Gaming Disorder Commentary on: Policy responses to problematic video game use: A systematic review of current measures and future possibilities (Király et al., 2018). J Behav Addict 2018;7:553–5. https://doi.org/10.1556/2006.7.2018.79.

[127]	Kyonka EGE, Schutte NS. Probability discounting and gambling: a meta-analysis. Addiction 2018;113:2173–81. https://doi.org/10.1111/add.14397.

[128]	Lai D, Johnson EC, Colbert S, Pandey G, Chan G, Bauer L, et al. Evaluating risk for alcohol use disorder: Polygenic risk scores and family history. Alcohol Clin Exp Res 2022;46:374–83. https://doi.org/10.1111/acer.14772.

[129]	Lane W, Sacco P, Downton K, Ludeman E, Levy L, Tracy JK. Child maltreatment and problem gambling: A systematic review. Child Abuse and Neglect 2016;58:24–38. https://doi.org/10.1016/j.chiabu.2016.06.003.

[130]	Latella D, Maggio MG, Andaloro A, Marchese D, Manuli A, Calabrò RS. Hypersexuality in neurological diseases: do we see only the tip of the iceberg? J Integr Neurosci 2021;20:477–87. https://doi.org/10.31083/j.jin2002051.

[131]	Leeman RF, Rowland BHP, Gebru NM, Potenza MN. Relationships among impulsive, addictive and sexual tendencies and behaviours: a systematic review of experimental and prospective studies in humans. Philos Trans R Soc Lond B Biol Sci 2019;374:20180129. https://doi.org/10.1098/rstb.2018.0129.

[132]	Legault MCB, Liu HZ, Balodis IM. Neuropsychological Constructs in Gaming Disorders: a Systematic Review. Current Behavioral Neuroscience Reports 2021;8:59–76. https://doi.org/10.1007/s40473-021-00230-z.

[133]	Lemenager T, Neissner M, Sabo T, Mann K, Kiefer F. “Who Am I” and “How Should I Be”: a Systematic Review on Self-Concept and Avatar Identification in Gaming Disorder. Curr Addict Rep 2020;7:166–93. https://doi.org/10.1007/s40429-020-00307-x.

[134]	Li L, Yu H, Liu Y, Meng Y-J, Li X-J, Zhang C, et al. Lower regional grey matter in alcohol use disorders: evidence from a voxel-based meta-analysis. BMC Psychiatry 2021;21:247. https://doi.org/10.1186/s12888-021-03244-9.

[135]	Lo CKM, Cho YW, Chen Q, Chan KL. Associations Between Different Types of Child Victimization and Gaming Disorder in Children and Adolescents: A Meta-Analysis. Trauma, Violence, and Abuse 2025. https://doi.org/10.1177/15248380251349781.

[136]	Loo JMY, Kraus SW, Potenza MN. A systematic review of gambling-related findings from the National Epidemiologic Survey on Alcohol and Related Conditions. Journal of Behavioral Addictions 2019;8:625–48. https://doi.org/10.1556/2006.8.2019.64.

[137]	Lopez-Quintero C, Pérez de los Cobos J, Hasin DS, Okuda M, Wang S, Grant BF, et al. Probability and predictors of transition from first use to dependence on nicotine, alcohol, cannabis, and cocaine: results of the National Epidemiologic Survey on Alcohol and Related Conditions (NESARC). Drug Alcohol Depend 2011;115:120–30. https://doi.org/10.1016/j.drugalcdep.2010.11.004.

[138]	Lorains FK, Cowlishaw S, Thomas SA. Prevalence of comorbid disorders in problem and pathological gambling: Systematic review and meta-analysis of population surveys. Addiction 2011;106:490–8. https://doi.org/10.1111/j.1360-0443.2010.03300.x.

[139]	Lui PP, Chmielewski M, Trujillo M, Morris J, Pigott TD. Linking Big Five Personality Domains and Facets to Alcohol (Mis)Use: A Systematic Review and Meta-Analysis. Alcohol Alcohol 2022;57:58–73. https://doi.org/10.1093/alcalc/agab030.

[140]	Luijten M, Machielsen MWJ, Veltman DJ, Hester R, de Haan L, Franken IHA. Systematic review of ERP and fMRI studies investigating inhibitory control and error processing in people with substance dependence and behavioural addictions. J Psychiatry Neurosci 2014;39:149–69. https://doi.org/10.1503/jpn.130052.

[141]	Luijten M, Schellekens AF, Kühn S, MacHielse MWJ, Sescousse G. Disruption of reward processing in addiction: An image-based meta-analysis of functional magnetic resonance imaging studies. JAMA Psychiatry 2017;74:387–98. https://doi.org/10.1001/jamapsychiatry.2016.3084.

[142]	Lyssenko L, Schmahl C, Bockhacker L, Vonderlin R, Bohus M, Kleindienst N. Dissociation in psychiatric disorders: A meta-analysis of studies using the dissociative experiences scale. American Journal of Psychiatry 2018;175:37–46. https://doi.org/10.1176/appi.ajp.2017.17010025.

[143]	MacLaren VV, Fugelsang JA, Harrigan KA, Dixon MJ. The personality of pathological gamblers: A meta-analysis. Clinical Psychology Review 2011;31:1057–67. https://doi.org/10.1016/j.cpr.2011.02.002.

[144]	Manfredi L, Accoto A, Couyoumdjian A, Conversi D. A Systematic Review of Genetic Polymorphisms Associated with Binge Eating Disorder. Nutrients 2021;13. https://doi.org/10.3390/nu13030848.

[145]	Männikkö N, Ruotsalainen H, Miettunen J, Pontes HM, Kääriäinen M. Problematic gaming behaviour and health-related outcomes: A systematic review and meta-analysis. Journal of Health Psychology 2020;25:67–81. https://doi.org/10.1177/1359105317740414.

[146]	Maraz A, Griffiths M, Demetrovics Z. The prevalence of compulsive buying: a meta-analysis. ADDICTION 2016;111:408–19. https://doi.org/10.1111/add.13223.

[147]	Marchetti D, Verrocchio MC, Porcelli P. Gambling problems and alexithymia: A systematic review. Brain Sciences 2019;9. https://doi.org/10.3390/brainsci9080191.

[148]	Marchica LA, Mills DJ, Derevensky JL, Montreuil TC. The Role of Emotion Regulation in Video Gaming and Gambling Disorder: A Systematic Review. Canadian Journal of Addiction 2019;10:19–29. https://doi.org/10.1097/CXA.0000000000000070.

[149]	Marciano L, Camerini A-L, Schulz PJ. Neuroticism in the digital age: A meta-analysis. Comput Hum Behav Rep 2020;2. https://doi.org/10.1016/j.chbr.2020.100026.

[150]	Mason TB, Lesser EL, Dolgon-Krutolow AR, Wonderlich SA, Smith KE. An updated transdiagnostic review of social cognition and eating disorder psychopathology. J Psychiatr Res 2021;143:602–27. https://doi.org/10.1016/j.jpsychires.2020.11.019.

[151]	McClelland J, Dalton B, Kekic M, Bartholdy S, Campbell IC, Schmidt U. A systematic review of temporal discounting in eating disorders and obesity: Behavioural and neuroimaging findings. Neurosci Biobehav Rev 2016;71:506–28. https://doi.org/10.1016/j.neubiorev.2016.09.024.

[152]	McDonald CE, Rossell SL, Phillipou A. The comorbidity of eating disorders in bipolar disorder and associated clinical correlates characterised by emotion dysregulation and impulsivity: A systematic review. Journal of Affective Disorders 2019;259:228–43. https://doi.org/10.1016/j.jad.2019.08.070.

[153]	McPhee MD, Hendershot CS. Meta-analysis of acute alcohol effects on response inhibition. Neurosci Biobehav Rev 2023;152:105274. https://doi.org/10.1016/j.neubiorev.2023.105274.

[154]	Meng Y-J, Deng W, Wang H-Y, Guo W-J, Li T, Lam C, et al. Reward pathway dysfunction in gambling disorder: A meta-analysis of functional magnetic resonance imaging studies. Behavioural Brain Research 2014;275:243–51. https://doi.org/10.1016/j.bbr.2014.08.057.

[155]	Meng Y, Deng W, Wang H, Guo W, Li T. The prefrontal dysfunction in individuals with Internet gaming disorder: A meta‐analysis of functional magnetic resonance imaging studies. Addiction Biology 2015;20:799–808. https://doi.org/10.1111/adb.12154.

[156]	Meque I, Dachew BA, Maravilla JC, Salom C, Alati R. Externalizing and internalizing symptoms in childhood and adolescence and the risk of alcohol use disorders in young adulthood: A meta-analysis of longitudinal studies. Aust N Z J Psychiatry 2019;53:965–75. https://doi.org/10.1177/0004867419844308.

[157]	Meyer C, Taranis L, Goodwin H, Haycraft E. Compulsive exercise and eating disorders. European Eating Disorders Review 2011;19:174–89. https://doi.org/10.1002/erv.1122.

[158]	Mihara S, Higuchi S. Cross‐sectional and longitudinal epidemiological studies of internet gaming disorder: A systematic review of the literature. Psychiatry and Clinical Neurosciences 2017;71:425–44. https://doi.org/10.1111/pcn.12532.

[159]	Mitchell E, Chohan H, Bestwick JP, Noyce AJ. Alcohol and Parkinson’s Disease: A Systematic Review and Meta-Analysis. J Parkinsons Dis 2022;12:2369–81. https://doi.org/10.3233/JPD-223522.

[160]	Moccia L, Pettorruso M, De Crescenzo F, De Risio L, di Nuzzo L, Martinotti G, et al. Neural correlates of cognitive control in gambling disorder: a systematic review of fMRI studies. Neuroscience and Biobehavioral Reviews 2017;78:104–16. https://doi.org/10.1016/j.neubiorev.2017.04.025.

[161]	Molde H, Moussavi Y, Kopperud ST, Erga AH, Hansen AL, Pallesen S. Impulse-control disorders in Parkinson’s Disease: A meta-analysis and review of case-control studies. Frontiers in Neurology 2018;9. https://doi.org/10.3389/fneur.2018.00330.

[162]	Montgomery-Graham S. Conceptualization and Assessment of Hypersexual Disorder: A Systematic Review of the Literature. Sex Med Rev 2017;5:146–62. https://doi.org/10.1016/j.sxmr.2016.11.001.

[163]	Monserrat Hernández M, Jiménez-Rodríguez D. Relationship of Genetic Polymorphisms and Microbial Composition with Binge Eating Disorder: A Systematic Review. Healthcare (Basel) 2024;12. https://doi.org/10.3390/healthcare12141441.

[164]	Moore KE, Oberleitner LMS, Zonana HV, Buchanan AW, Pittman BP, Verplaetse TL, et al. Psychiatric Disorders and Crime in the US Population: Results From the National Epidemiologic Survey on Alcohol and Related Conditions Wave III. J Clin Psychiatry 2019;80:18m12317. https://doi.org/10.4088/JCP.18m12317.

[165]	Moore LH III, Grubbs JB. Gambling Disorder and comorbid PTSD: A systematic review of empirical research. Addictive Behaviors 2021;114. https://doi.org/10.1016/j.addbeh.2020.106713.

[166]	Mora-Salgueiro J, García-Estela A, Hogg B, Angarita-Osorio N, Amann BL, Carlbring P, et al. The Prevalence and Clinical and Sociodemographic Factors of Problem Online Gambling: A Systematic Review. Journal of Gambling Studies 2021;37:899–926. https://doi.org/10.1007/s10899-021-09999-w.

[167]	Morkeviciute M, Endriulaitiene A, Poskus M. Understanding the etiology of workaholism: The results of the systematic review and meta-analysis. JOURNAL OF WORKPLACE BEHAVIORAL HEALTH 2021;36:351–72. https://doi.org/10.1080/15555240.2021.1968882.

[168]	Nakum S, Cavanna AE. The prevalence and clinical characteristics of hypersexuality in patients with Parkinson’s disease following dopaminergic therapy: A systematic literature review. Parkinsonism & Related Disorders 2016;25:10–6. https://doi.org/10.1016/j.parkreldis.2016.02.017.

[169]	Nazar BP, Bernardes C, Peachey G, Sergeant J, Mattos P, Treasure J. The risk of eating disorders comorbid with attention‐deficit/hyperactivity disorder: A systematic review and meta‐analysis. International Journal of Eating Disorders 2016;49:1045–57. https://doi.org/10.1002/eat.22643.

[170]	Nicholls W, Devonport TJ, Blake M. The association between emotions and eating behaviour in an obese population with binge eating disorder. Obes Rev 2016;17:30–42. https://doi.org/10.1111/obr.12329.

[171]	Nielsen P, Favez N, Liddle H, Rigter H. Linking parental mediation practices to adolescents’ problematic online screen use: A systematic literature review. JOURNAL OF BEHAVIORAL ADDICTIONS 2019;8:649–63. https://doi.org/10.1556/2006.8.2019.61.

[172]	Nielsen P, Favez N, Rigter H. Parental and Family Factors Associated with Problematic Gaming and Problematic Internet Use in Adolescents: a Systematic Literature Review. CURRENT ADDICTION REPORTS 2020;7:365–86. https://doi.org/10.1007/s40429-020-00320-0.

[173]	Niu X, Gao X, Zhang M, Yang Z, Yu M, Wang W, et al. Meta-analysis of structural and functional brain alterations in internet gaming disorder. Front Psychiatry 2022;13:1029344. https://doi.org/10.3389/fpsyt.2022.1029344.

[174]	Nordmyr J, Forsman AK. A systematic review of psychosocial risks for gambling and problem gambling in the Nordic countries. Health, Risk and Society 2020;22:266–90. https://doi.org/10.1080/13698575.2020.1796929.

[175]	Nowak DE. A Meta-analytical Synthesis and Examination of Pathological and Problem Gambling Rates and Associated Moderators Among College Students, 1987–2016. Journal of Gambling Studies 2018;34:465–98. https://doi.org/10.1007/s10899-017-9726-y.

[176]	Nowakowski ME, McFarlane T, Cassin S. Alexithymia and eating disorders: a critical review of the literature. Journal of Eating Disorders 2013;1:21. https://doi.org/10.1186/2050-2974-1-21.

[177]	Nuske J, Nuske L, Stevens MWR, Billieux J, Delfabbro PH, Hides L, et al. The association between gaming disorder and impulsivity: A systematic review and meta-analysis. Aust N Z J Psychiatry 2026;60:26–41. https://doi.org/10.1177/00048674251388779.

[178]	Nuyens F, Kuss DJ, Lopez-Fernandez O, Griffiths MD. The experimental analysis of problematic video gaming and cognitive skills: A systematic review. Journal de Thérapie Comportementale et Cognitive 2017;27:110–7. https://doi.org/10.1016/j.jtcc.2017.05.001.

[179]	Olguin P, Fuentes M, Gabler G, Guerdjikova AI, Keck PE Jr, McElroy SL. Medical comorbidity of binge eating disorder. Eating Weight Disord 2017;22:13–26. https://doi.org/10.1007/s40519-016-0313-5.

[180]	Palmisano GL, Innamorati M, Vanderlinden J. Life adverse experiences in relation with obesity and binge eating disorder: A systematic review. Journal of Behavioral Addictions 2016;5:11–31. https://doi.org/10.1556/2006.5.2016.018.

[181]	Paulus FW, Ohmann S, von Gontard A, Popow C. Internet gaming disorder in children and adolescents: A systematic review. Developmental Medicine & Child Neurology 2018;60:645–59. https://doi.org/10.1111/dmcn.13754.

[182]	Pelzer M, Brockmeyer T, Tuschen-Caffier B, Werthmann J. Food and body-related attentional biases in children and adolescents with eating disorder symptoms, overweight and obesity: a systematic review. J Eat Disord 2025;14:9. https://doi.org/10.1186/s40337-025-01459-9.

[183]	Petrescu T-C, Błachnio A, Enea V. Internet gaming disorder in children and adolescents: A systematic review of familial protective and risk factors. Addict Behav 2025;167:108345. https://doi.org/10.1016/j.addbeh.2025.108345.

[184]	Pettorruso M, Zoratto F, Miuli A, De Risio L, Santorelli M, Pierotti A, et al. Exploring dopaminergic transmission in gambling addiction: A systematic translational review. Neuroscience and Biobehavioral Reviews 2020;119:481–511. https://doi.org/10.1016/j.neubiorev.2020.09.034.

[185]	Pinna F, Dell’Osso B, Di Nicola M, Janiri L, Altamura AC, Carpiniello B, et al. Behavioural addictions and the transition from DSM-IV-TR to DSM-5. Journal of Psychopathology 2015;21:380–9.

[186]	Pirwani N, Szabo A. Exercise Addiction: A Systematic Review of Neuroimaging Evidence. Current Addiction Reports 2025;12. https://doi.org/10.1007/s40429-025-00693-0.

[187]	Prefit A-B, Cândea DM, Szentagotai-Tătar A. Emotion regulation across eating pathology: A meta-analysis. Appetite 2019;143:104438. https://doi.org/10.1016/j.appet.2019.104438.

[188]	Prunell-Castañé A, Jurado MÁ, García-García I. Clinical binge eating, but not uncontrolled eating, is associated with differences in executive functions: Evidence from meta-analytic findings. Addict Behav Rep 2021;13. https://doi.org/10.1016/j.abrep.2020.100337.

[189]	Punia K, DeVillaer M, MacKillop J, Balodis IM. Understanding the Overlap Between Cannabis Use and Gambling Behaviour: A Systematic Review of Empirical Findings and Consideration of Policy Implications. Current Addiction Reports 2021;8:35–56. https://doi.org/10.1007/s40429-020-00323-x.

[190]	Qin K, Zhang F, Chen T, Li L, Li W, Suo X, et al. Shared gray matter alterations in individuals with diverse behavioral addictions: A voxel-wise meta-analysis. Journal of Behavioral Addictions 2020;9:44–57. https://doi.org/10.1556/2006.2020.00006.

[191]	Quaglieri A, Mari E, Boccia M, Piccardi L, Guariglia C, Giannini AM. Brain network underlying executive functions in gambling and alcohol use disorders: An activation likelihood estimation meta-analysis of FMRI studies. Brain Sciences 2020;10:1–19. https://doi.org/10.3390/brainsci10060353.

[192]	Raimo S, Cropano M, Trojano L, Santangelo G. The neural basis of gambling disorder: An activation likelihood estimation meta-analysis. Neuroscience and Biobehavioral Reviews 2021;120:279–302. https://doi.org/10.1016/j.neubiorev.2020.11.027.

[193]	Ralph-Nearman C, Achee M, Lapidus R, Stewart JL, Filik R. A systematic and methodological review of attentional biases in eating disorders: Food, body, and perfectionism. Brain Behav 2019;9:e01458. https://doi.org/10.1002/brb3.1458.

[194]	Raybould JN, Larkin M, Tunney RJ. Is there a health inequality in gambling related harms? A systematic review. BMC Public Health 2021;21. https://doi.org/10.1186/s12889-021-10337-3.

[195]	Refahi Z, Arian Namazi S, King DL, Sadeghi S. Attentional Bias in Gaming Addiction: A Systematic Review of Behavioral Studies. Current Addiction Reports 2025;12. https://doi.org/10.1007/s40429-025-00691-2.

[196]	Richard J, Fletcher E, Boutin S, Derevensky J, Temcheff C. Conduct problems and depressive symptoms in association with problem gambling and gaming: A systematic review. Journal of Behavioral Addictions 2020;9:497–533. https://doi.org/10.1556/2006.2020.00045.

[197]	Rizk M, Mattar L, Kern L, Berthoz S, Duclos J, Viltart O, et al. Physical Activity in Eating Disorders: A Systematic Review. Nutrients 2020;12:183. https://doi.org/10.3390/nu12010183.

[198]	Roerecke M, Rehm J. Cause-specific mortality risk in alcohol use disorder treatment patients: a systematic review and meta-analysis. Int J Epidemiol 2014;43:906–19. https://doi.org/10.1093/ije/dyu018.

[199]	Rogier G, Beomonte Zobel S, Marini A, Camponeschi J, Velotti P. Gambling disorder and dissociative features: A systematic review and meta-analysis. Psychology of Addictive Behaviors 2021;35:247–62. https://doi.org/10.1037/adb0000693.

[200]	Rogier G, Beomonte Zobel S, Morganti W, Ponzoni S, Velotti P. Metacognition in gambling disorder: A systematic review and meta-analysis. Addictive Behaviors 2021;112. https://doi.org/10.1016/j.addbeh.2020.106600.

[201]	Rooke SE, Hine DW, Thorsteinsson EB. Implicit cognition and substance use: a meta-analysis. Addict Behav 2008;33:1314–28. https://doi.org/10.1016/j.addbeh.2008.06.009.

[202]	Ropovik I, Martončik M, Babinčák P, Baník G, Vargová L, Adamkovič M. Risk and protective factors for (internet) gaming disorder: A meta-analysis of pre-COVID studies. Addict Behav 2023;139:107590. https://doi.org/10.1016/j.addbeh.2022.107590.

[203]	Şalvarlı Şİ, Griffiths MD. Internet Gaming Disorder and Its Associated Personality Traits: A Systematic Review Using PRISMA Guidelines. Int J Ment Health Addict 2021;19:1420–42. https://doi.org/10.1007/s11469-019-00081-6.

[204]	Şalvarlı Şİ, Griffiths MD. The Association Between Internet Gaming Disorder and Impulsivity: A Systematic Review of Literature. Int J Ment Health Addict 2022;20:92–118. https://doi.org/10.1007/s11469-019-00126-w.

[205]	Schacht JP, Anton RF, Myrick H. Functional neuroimaging studies of alcohol cue reactivity: a quantitative meta-analysis and systematic review. Addict Biol 2013;18:121–33. https://doi.org/10.1111/j.1369-1600.2012.00464.x.

[206]	Schettler L, Thomasius R, Paschke K. Neural correlates of problematic gaming in adolescents: A systematic review of structural and functional magnetic resonance imaging studies. Addiction Biology 2022;27. https://doi.org/10.1111/adb.13093.

[207]	Schneider LA, King DL, Delfabbro PH. Family factors in adolescent problematic Internet gaming: A systematic review. J Behav Addict 2017;6:321–33. https://doi.org/10.1556/2006.6.2017.035.

[208]	Schultz K, Hook JN, Davis DE, Penberthy JK, Reid RC. Nonparaphilic hypersexual behavior and depressive symptoms: A meta-analytic review of the literature. Journal of Sex & Marital Therapy 2014;40:477–87. https://doi.org/10.1080/0092623X.2013.772551.

[209]	Senra H, Gaglianone CG, McPherson S, Unterrainer H. Prevalence of personality disorders in adults with binge eating disorder-A systematic review and Bayesian meta-analysis. Obes Rev 2024;25:e13669. https://doi.org/10.1111/obr.13669.

[210]	Sharman S, Butler K, Roberts A. Psychosocial risk factors in disordered gambling: A descriptive systematic overview of vulnerable populations. Addictive Behaviors 2019;99. https://doi.org/10.1016/j.addbeh.2019.106071.

[211]	Sheehan DV, Herman BK. The psychological and medical factors associated with untreated binge eating disorder. Prim Care Companion J Clin Psych 2015;17. https://doi.org/10.4088/PCC.14r01732.

[212]	Simon-Grima J, Estrada-Marcen N, Montero-Marin J. Exercise addiction measure through the Exercise Addiction Inventory (EAI) and health in habitual exercisers. A systematic review and meta-analysis. ADICCIONES 2019;31:233–49. https://doi.org/10.20882/adicciones.990.

[213]	Slavin MN, Scoglio AAJ, Blycker GR, Potenza MN, Kraus SW. Child Sexual Abuse and Compulsive Sexual Behavior: A Systematic Literature Review. Curr Addict Rep 2020;7:76–88. https://doi.org/10.1007/s40429-020-00298-9.

[214]	Slavin MN, Hewage S, Roy S, Kraus SW. Child Sexual Abuse and Compulsive Sexual Behavior: An Updated Systematic Review of Key Findings and Moderators. Current Addiction Reports 2025;12. https://doi.org/10.1007/s40429-025-00658-3.

[215]	Smith KE, Mason TB, Johnson JS, Lavender JM, Wonderlich SA. A systematic review of reviews of neurocognitive functioning in eating disorders: The state-of-the-literature and future directions. Int J Eat Disord 2018;51:798–821. https://doi.org/10.1002/eat.22929.

[216]	Sohn MN, Dimitropoulos G, Ramirez A, McPherson C, Anderson A, Munir A, et al. Non-suicidal self-injury, suicidal thoughts and behaviors in individuals with an eating disorder relative to healthy and psychiatric controls: A systematic review and meta-analysis. Int J Eat Disord 2023;56:501–15. https://doi.org/10.1002/eat.23880.

[217]	Sol-Gámez J, Conde G, Vázquez-Martínez A, Villanueva-Blasco V. Prevalence and Determinants of Compulsive Buying: A Systematic Review with Preventive Implications. ACCIONES E INVESTIGACIONES SOCIALES 2025:66–96. https://doi.org/10.26754/ojs_ais/accionesinvestigsoc.20254610968.

[218]	Soldati L, Bianchi-Demicheli F, Schockaert P, Köhl J, Bolmont M, Hasler R, et al. Association of ADHD and hypersexuality and paraphilias. Psychiatry Res 2021;295:113638. https://doi.org/10.1016/j.psychres.2020.113638.

[219]	Solmi M, Veronese N, Sergi G, Luchini C, Favaro A, Santonastaso P, et al. The association between smoking prevalence and eating disorders: A systematic review and meta‐analysis. Addiction 2016;111:1914–22. https://doi.org/10.1111/add.13457.

[220]	Spindler C, Mallien L, Trautmann S, Alexander N, Muehlhan M. A coordinate-based meta-analysis of white matter alterations in patients with alcohol use disorder. Transl Psychiatry 2022;12:40. https://doi.org/10.1038/s41398-022-01809-0.

[221]	Spurrier M, Blaszczynski A. Risk Perception in Gambling: A Systematic Review. Journal of Gambling Studies 2014;30:253–76. https://doi.org/10.1007/s10899-013-9371-z.

[222]	Starcke K, Antons S, Trotzke P, Brand M. Cue-reactivity in behavioral addictions: A meta-analysis and methodological considerations. Journal of Behavioral Addictions 2018;7:227–38. https://doi.org/10.1556/2006.7.2018.39.

[223]	Stellern J, Xiao KB, Grennell E, Sanches M, Gowin JL, Sloan ME. Emotion regulation in substance use disorders: a systematic review and meta-analysis. Addiction 2023;118:30–47. https://doi.org/10.1111/add.16001.

[224]	Stephan RA, Alhassoon OM, Allen KE, Wollman SC, Hall M, Thomas WJ, et al. Meta-analyses of clinical neuropsychological tests of executive dysfunction and impulsivity in alcohol use disorder. American Journal of Drug and Alcohol Abuse 2017;43:24–43. https://doi.org/10.1080/00952990.2016.1206113.

[225]	Stevens MWR, Dorstyn D, Delfabbro PH, King DL. Global prevalence of gaming disorder: A systematic review and meta-analysis. Australian and New Zealand Journal of Psychiatry 2021;55:553–68. https://doi.org/10.1177/0004867420962851.

[226]	Stojek MMK, MacKillop J. Relative reinforcing value of food and delayed reward discounting in obesity and disordered eating: A systematic review. Clinical Psychology Review 2017;55:1–11. https://doi.org/10.1016/j.cpr.2017.04.007.

[227]	Stojek M, Shank LM, Vannucci A, Bongiorno DM, Nelson EE, Waters AJ, et al. A systematic review of attentional biases in disorders involving binge eating. Appetite 2018;123:367–89. https://doi.org/10.1016/j.appet.2018.01.019.

[228]	Strømme R, Børstad KH, Rø AE, Erevik EK, Sagoe D, Chegeni R, et al. The Relationship Between Gambling Problems and the Five-Factor Model of Personality: A Systematic Review and Meta-Analysis. Frontiers in Psychiatry 2021;12. https://doi.org/10.3389/fpsyt.2021.740235.

[229]	Subramaniam M, Wang P, Soh P, Vaingankar JA, Chong SA, Browning CJ, et al. Prevalence and determinants of gambling disorder among older adults: A systematic review. Addictive Behaviors 2015;41:199–209. https://doi.org/10.1016/j.addbeh.2014.10.007.

[230]	Sugaya N, Shirasaka T, Takahashi K, Kanda H. Bio-psychosocial factors of children and adolescents with internet gaming disorder: a systematic review. BIOPSYCHOSOCIAL MEDICINE 2019;13. https://doi.org/10.1186/s13030-019-0144-5.

[231]	Suresh N, Kantipudi SJ, Ramu D, Muniratnam SK, Venkatesan V. Association between opioid and dopamine receptor gene polymorphisms OPRM1 rs1799971, DAT VNTR 9-10 repeat allele, DRD1 rs4532 and DRD2 rs1799732 and alcohol dependence: an ethnicity oriented meta-analysis. Pharmacogenet Genomics 2023;33:139–52. https://doi.org/10.1097/FPC.0000000000000502.

[232]	Sussman S, Lisha N, Griffiths M. Prevalence of the addictions: A problem of the majority or the minority? Evaluation and the Health Professions 2011;34:3–56. https://doi.org/10.1177/0163278710380124.

[233]	Szinay D, Tombor I, Garnett C, Boyt N, West R. Associations between self-esteem and smoking and excessive alcohol consumption in the UK: A cross-sectional study using the BBC UK Lab database. Addict Behav Rep 2019;10:100229. https://doi.org/10.1016/j.abrep.2019.100229.

[234]	Tael-Öeren M, Naughton F, Sutton S. The relationship between parental attitudes and children’s alcohol use: a systematic review and meta-analysis. Addiction 2019;114:1527–46. https://doi.org/10.1111/add.14615.

[235]	Theisejans J, Thomas TA, Oueslati FBV, Kessling A, Wegmann E, Müller A, et al. Affective and Cognitive Functions, Impulsivity and Compulsivity in Problematic Social Network Use and in Compulsive Buying-Shopping Disorder: A Systematic Review. Current Addiction Reports 2025;12. https://doi.org/10.1007/s40429-025-00688-x.

[236]	Theule J, Hurl KE, Cheung K, Ward M, Henrikson B. Exploring the Relationships Between Problem Gambling and ADHD: A Meta-Analysis. Journal of Attention Disorders 2019;23:1427–37. https://doi.org/10.1177/1087054715626512.

[237]	Thiebaut S, Godart N, Radon L, Courtet P, Guillaume S. Crossed prevalence results between subtypes of eating disorder and bipolar disorder: A systematic review of the literature. L’Encéphale: Revue de Psychiatrie Clinique Biologique et Thérapeutique 2019;45:60–73. https://doi.org/10.1016/j.encep.2018.06.001.

[238]	Thomas TA, Joshi M, Trotzke P, Steins-Loeber S, Müller A. Cognitive Functions in Compulsive Buying-Shopping Disorder: a Systematic Review. Current Behavioral Neuroscience Reports 2023;10:1–19. https://doi.org/10.1007/s40473-023-00255-6.

[239]	Trott M, Jackson SE, Firth J, Fisher A, Johnstone J, Mistry A, et al. Exercise Addiction Prevalence and Correlates in the Absence of Eating Disorder Symptomology: A Systematic Review and Meta-analysis. J Addict Med 2020;14:e321–9. https://doi.org/10.1097/ADM.0000000000000664.

[240]	Tse S, Hong S-I, Wang C-W, Cunningham-Williams RM. Gambling behavior and problems among older adults: A systematic review of empirical studies. Journals of Gerontology - Series B Psychological Sciences and Social Sciences 2012;67 B:639–52. https://doi.org/10.1093/geronb/gbs068.

[241]	Unsworth DJ, Mathias JL. Traumatic brain injury and alcohol/substance abuse: A Bayesian meta-analysis comparing the outcomes of people with and without a history of abuse. J Clin Exp Neuropsychol 2017;39:547–62. https://doi.org/10.1080/13803395.2016.1248812.

[242]	van Timmeren T, Daams JG, van Holst RJ, Goudriaan AE. Compulsivity-related neurocognitive performance deficits in gambling disorder: A systematic review and meta-analysis. Neuroscience and Biobehavioral Reviews 2018;84:204–17. https://doi.org/10.1016/j.neubiorev.2017.11.022.

[243]	Van Tu P, Ngoc TU, Nguyen PL, Thuong NH, Van Tuan N, Van Hung N, et al. The impact of sexual harassment on obesity in female adolescents: An update and perspective to control. Diabetes Metab Syndr Clin Res Rev 2020;14:1931–9. https://doi.org/10.1016/j.dsx.2020.09.036.

[244]	Vandenberg B, Livingstone C, Carter A, O’Brien K. Gambling and homelessness: A systematic review and meta-analysis of prevalence. Addictive Behaviors 2022;125. https://doi.org/10.1016/j.addbeh.2021.107151.

[245]	Varo C, Murru A, Salagre E, Jiménez E, Solé B, Montejo L, et al. Behavioral addictions in bipolar disorders: A systematic review. European Neuropsychopharmacology 2019;29:76–97. https://doi.org/10.1016/j.euroneuro.2018.10.012.

[246]	Velotti P, Rogier G, Beomonte Zobel S, Billieux J. Association between gambling disorder and emotion (dys)regulation: A systematic review and meta-analysis. Clinical Psychology Review 2021;87. https://doi.org/10.1016/j.cpr.2021.102037.

[247]	Verplaetse TL, Peltier MR, Roberts W, Pittman B, McKee SA. Gender and past year serious psychological distress are associated with past year AUD: Time-varying results from the National Survey on Drug Use and Health (NSDUH; 2008-2017). Addict Behav 2021;116:106815. https://doi.org/10.1016/j.addbeh.2020.106815.

[248]	Waltmann M, Herzog N, Horstmann A, Deserno L. Loss of control over eating: A systematic review of task based research into impulsive and compulsive processes in binge eating. Neuroscience and Biobehavioral Reviews 2021;129:330–50. https://doi.org/10.1016/j.neubiorev.2021.07.016.

[249]	Wang X, Yang X, Tao T, Dong D, Yu D. The association between exercise addiction and mental health problems: A systematic review and meta-analysis. J Affect Disord 2026;393:120026. https://doi.org/10.1016/j.jad.2025.120026.

[250]	Weinsztok S, Brassard S, Balodis I, Martin LE, Amlung M. Delay Discounting in Established and Proposed Behavioral Addictions: A Systematic Review and Meta-Analysis. Frontiers in Behavioral Neuroscience 2021;15. https://doi.org/10.3389/fnbeh.2021.786358.

[251]	Wéry A, Billieux J. Problematic cybersex: Conceptualization, assessment, and treatment. Addict Behav 2017;64:238–46. https://doi.org/10.1016/j.addbeh.2015.11.007.

[252]	Westwood H, Kerr-Gaffney J, Stahl D, Tchanturia K. Alexithymia in eating disorders: Systematic review and meta-analyses of studies using the Toronto Alexithymia Scale. Journal of Psychosomatic Research 2017;99:66–81. https://doi.org/10.1016/j.jpsychores.2017.06.007.

[253]	Wilfley DE, Citrome L, Herman BK. Characteristics of binge eating disorder in relation to diagnostic criteria. Neuropsychiatr Dis Treat 2016;12:2213–23. https://doi.org/10.2147/NDT.S107777.

[254]	Wu M, Hartmann M, Skunde M, Herzog W, Friederich H-C. Inhibitory control in bulimic-type eating disorders: a systematic review and meta-analysis. PLoS One 2013;8:e83412. https://doi.org/10.1371/journal.pone.0083412.

[255]	Wu M, Brockmeyer T, Hartmann M, Skunde M, Herzog W, Friederich H-C. Set-shifting ability across the spectrum of eating disorders and in overweight and obesity: A systematic review and meta-analysis. Psychological Medicine 2014;44:3365–85. https://doi.org/10.1017/S0033291714000294.

[256]	Wu M, Brockmeyer T, Hartmann M, Skunde M, Herzog W, Friederich H. Reward-related decision making in eating and weight disorders: A systematic review and meta-analysis of the evidence from neuropsychological studies. NEUROSCIENCE AND BIOBEHAVIORAL REVIEWS 2016;61:177–96. https://doi.org/10.1016/j.neubiorev.2015.11.017.

[257]	Xuan Y-H, Li S, Tao R, Chen J, Rao L-L, T. Wang X, et al. Genetic and environmental influences on gambling: A meta-analysis of twin studies. Frontiers in Psychology 2017;8. https://doi.org/10.3389/fpsyg.2017.02121.

[258]	Xue R, Li J, Yang H. The hemispheric differences in prefrontal function of Internet game disorder and non-Internet game disorder: an activation likelihood estimation meta-analysis. Cereb Cortex 2025;35:bhae493. https://doi.org/10.1093/cercor/bhae493.

[259]	Yang X, Tian F, Zhang H, Zeng J, Chen T, Wang S, et al. Cortical and subcortical gray matter shrinkage in alcohol-use disorders: a voxel-based meta-analysis. Neurosci Biobehav Rev 2016;66:92–103. https://doi.org/10.1016/j.neubiorev.2016.03.034.

[260]	Yao Y-W, Liu L, Ma S-S, Shi X-H, Zhou N, Zhang J-T, et al. Functional and structural neural alterations in Internet gaming disorder: A systematic review and meta-analysis. Neuroscience and Biobehavioral Reviews 2017;83:313–24. https://doi.org/10.1016/j.neubiorev.2017.10.029.

[261]	Yao Y, Zhang J, Fang X, Liu L, Potenza M. Reward-related decision-making deficits in internet gaming disorder: a systematic review and meta-analysis. ADDICTION 2022;117:19–32. https://doi.org/10.1111/add.15518.

[262]	Yazdi-Feyzabadi V, Mehrolhassani MH, Zolala F, Haghdoost A, Oroomiei N. Determinants of risky sexual practice, drug abuse and alcohol consumption in adolescents in Iran: a systematic literature review. Reprod Health 2019;16:115. https://doi.org/10.1186/s12978-019-0779-5.

[263]	Young S, Rhodes P, Touyz S, Hay P. The relationship between obsessive-compulsive personality disorder traits, obsessive-compulsive disorder and excessive exercise in patients with anorexia nervosa: a systematic review. J Eat Disord 2013;1:16. https://doi.org/10.1186/2050-2974-1-16.

[264]	Yu L, Ma CMS. Youth Gambling in Hong Kong: Prevalence, Psychosocial Correlates, and Prevention. Journal of Adolescent Health 2019;64:S44–51. https://doi.org/10.1016/j.jadohealth.2018.09.019.

[265]	Yu J, Abdullah MFIL, Mansor NS. EEG components of inhibitory control ability in internet gaming disorder: A systematic review and meta-analysis of randomized controlled trials. Brain Behav 2024;14:e3388. https://doi.org/10.1002/brb3.3388.

[266]	Zeng J, You L, Yang F, Luo Y, Yu S, Yan J, et al. A meta-analysis of the neural substrates of monetary reward anticipation and outcome in alcohol use disorder. Hum Brain Mapp 2023;44:2841–61. https://doi.org/10.1002/hbm.26249.

[267]	Zeng J, You L, Sheng H, Luo Y, Yang X. The differential neural substrates for reward choice under gain-loss contexts and risk in alcohol use disorder: Evidence from a voxel-based meta-analysis. Drug Alcohol Depend 2023;248:109912. https://doi.org/10.1016/j.drugalcdep.2023.109912.

[268]	Zheng H, Hu Y, Wang Z, Wang M, Du X, Dong G. Meta-analyses of the functional neural alterations in subjects with Internet gaming disorder: Similarities and differences across different paradigms. PROGRESS IN NEURO-PSYCHOPHARMACOLOGY & BIOLOGICAL PSYCHIATRY 2019;94. https://doi.org/10.1016/j.pnpbp.2019.109656.

[269]	Zheng C, Liu K, Liang L, Yuan T, Li H, Qing Y, et al. Revisiting the relationship between adolescent internet gaming disorder and depression: A systematic review and meta-analysis. Adolescent Research Review 2025. https://doi.org/10.1007/s40894-025-00275-1.

[270]	Zheng H, Dong C, Wan Y, Shen H, Li S, Ji S, et al. Disruptions of white matter in adolescents with internet game disorder: Evidence from seed-based d mapping. Brain Imaging and Behavior 2025;19:1038–47. https://doi.org/10.1007/s11682-025-01033-6.

[271]	Zhuang X, Zhang Y, Tang X, Ng TK, Lin J, Yang X. Longitudinal modifiable risk and protective factors of internet gaming disorder: A systematic review and meta-analysis. J Behav Addict 2023;12:375–92. https://doi.org/10.1556/2006.2023.00017.
